# Supplementary material for: Statistical Properties of Lasso-Shape Polymers and Their Implications for Complex Lasso Proteins Function
Source: Polymers (Basel). 2019 Apr 17;11(4):707. doi: 10.3390/polym11040707 (PMC6523798; doi:10.3390/polym11040707)
Supplement: Supplementary file 1 [file polymers-11-00707-s001.pdf]

Statistical properties of lasso polymers and complex lasso proteins.

Supplementary Material

Pawel Dabrowski-Tumanski, Bartosz Gren, and Joanna I. Sulkowska

Contents

S1 Validation of the method of generating closed loops **S2**
S1.1 Acceptation rate test S2
S1.2 Curvature test S2
S1.3 HOMFLY-PT polynomial occurence test S2

S2 Statistical probabililty of complex lasso occurence **S3**
S2.1 Probability of trivial lassos S3

S3 Shape parameters of lasso loop **S5**
S3.1 Mean radius of gyration, distension, asphericity, and prolatness S5

S4 Protein data **S7**
S4.1 Scaling of the area of minimal surface in proteins. S7
S4.2 Calculation of the expected number of non-trivial proteins S7
S4.3 Non-trivial lassos S8
S4.4 List of proteins structures used S20

S1 Validation of the method of generating closed loops

Validation analogous to original paper [1] was done. Tests confirm correct reconstruction of method.

S1.1 Acceptation rate test

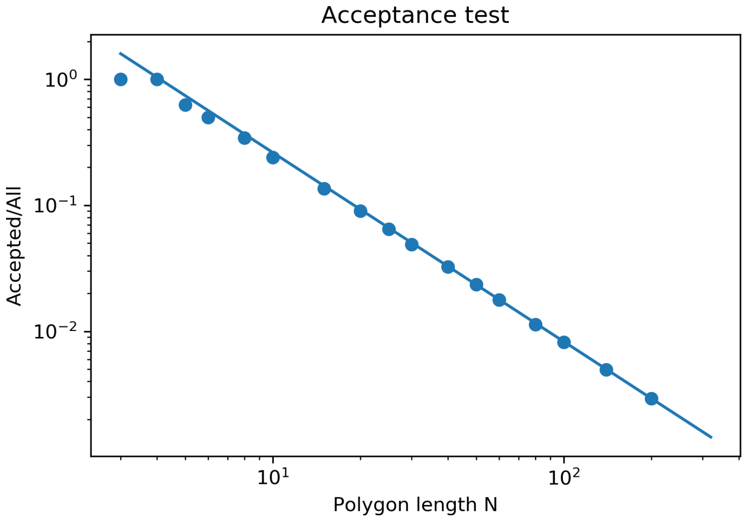

Figure S1: Many structures are rejected during generation because they won't satisfy algorithm assumptions. Then we can calculate ratio of accepted structures to the sum of accepted and rejected. The acceptance ratio fit to the distribution $p_N \sim 6\sqrt{6/\pi}N^{-3/2}$ [1] with N denoting the polygon length.

S1.2 Curvature test

**Source**	Number of tries	31-gon	32-gon
Theory	—	49.912	51.482
Original work [1]	60,000	49.902	51.475
This work	60,000	49,899	51.514

Table S1: Average total curvature for 31- and 32-gons were calculated. Total curvature is a sum of all turning angles.

S1.3 HOMFLY-PT polynomial occurence test

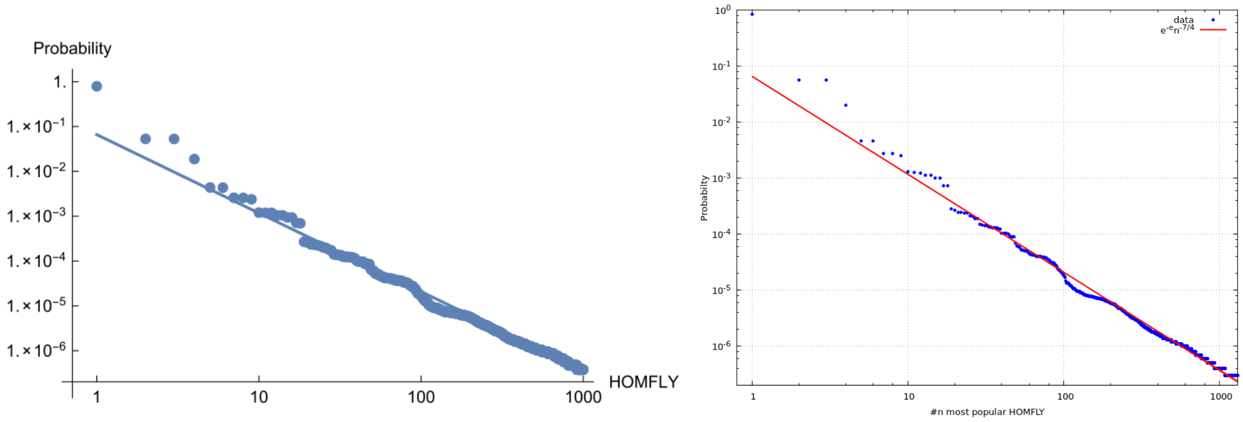

Figure S2: The probability of occurence of distinct HOMFLY-PT polynomials, starting from the most common. Left panel: original work [1], right panel - this work. The fit was done to the function $e^{-e}n^{-7/5}$ with n being the number of the polynomial in the polynomial list sorted according to the number of occurrences.

S2 Statistical probabililty of complex lasso occurence

S2.1 Probability of trivial lassos

The surface of probability of trivial lassos was fitted with:

$$P(l,t) = A_\infty + c_{\alpha;t}\exp(-\alpha_t t) + c_{\alpha;l}\exp(-\alpha_l l) + c_{\beta;t}\exp(-\beta_t t) + c_{\beta;l}\exp(-\beta_l l) \quad (1)$$

With the following parameters obtained:

Variable	Fitted value	Fiting assymptotic error
$P_{L_0;\infty}$	0.19682	$\pm$ 0.004581 (2.327%)
$c_{\alpha;t}$	0.241859	$\pm$ 0.01228 (5.077%)
α_t	0.0700785	$\pm$ 0.006895 (9.839%)
$c_{\alpha;l}$	0.38344	$\pm$ 0.009612 (2.507%)
α_l	0.0484631	$\pm$0.002643 (5.453%)
$c_{\beta;t}$	0.107235	$\pm$ 0.007373 (6.875%)
β_t	0.00835938	$\pm$ 0.001041 (12.45%)
$c_{\beta;l}$	0.309337	$\pm$ 0.008828 (2.854%)
β_l	0.00671983	$\pm$ 0.0003902 (5.807%)

Table S2: The fit parameters for the surface fit of the probability of trivial lasso.

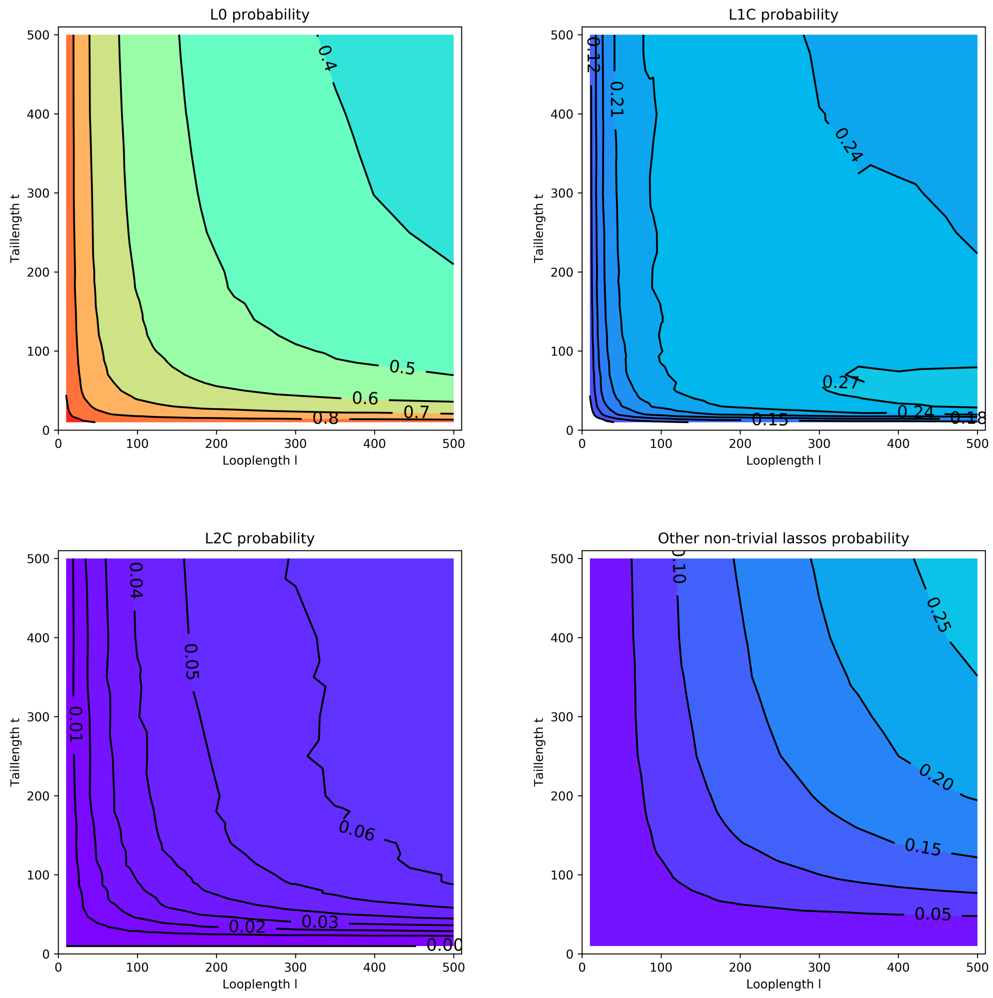

Figure S3: Contour plots of different lasso types in the space of tail and loop length.

In each case the fit was done with the function:

$$P_{L_0}(N) = A_\infty + c_\alpha \exp(-\alpha N) + c_\beta \exp(-\beta N) \quad (2)$$

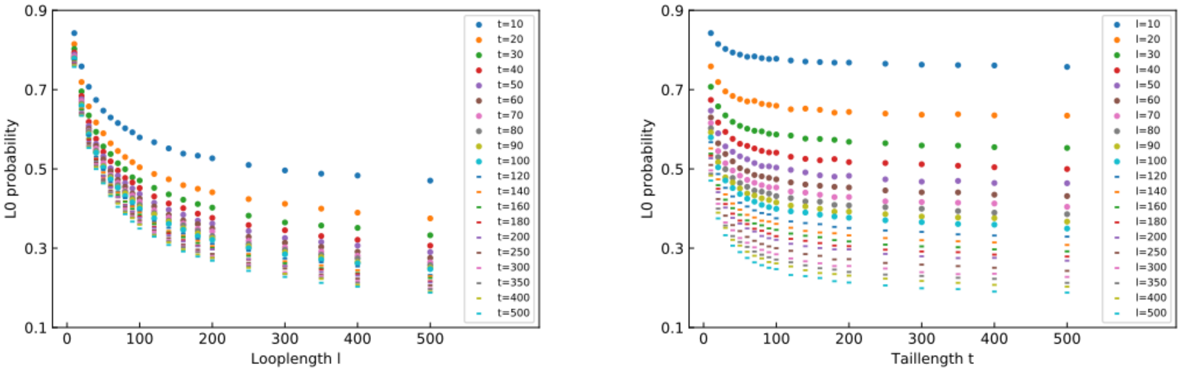

Figure S4: Probability of trivial lasso vs loop length (left panel) or vs tail length (right panel).

**Fixed length**	Fixed tail length					Fixed loop length				
	A_∞	c_α	α	c_β	β	A_∞	c_α	α	c_β	β
10	0.465	0.224	0.278	0.00666	0.0517	0.758	0.0367	0.095	0.00661	0.0632
20	0.364	0.242	0.336	0.00566	0.0429	0.634	0.0670	0.144	0.01019	0.0806
30	0.325	0.269	0.348	0.00631	0.0456	0.551	0.0712	0.170	0.00667	0.0644
40	0.298	0.286	0.357	0.00639	0.0470	0.502	0.0847	0.185	0.00818	0.0685
50	0.283	0.290	0.368	0.00655	0.0472	0.463	0.0983	0.198	0.00915	0.0747
60	0.266	0.288	0.373	0.00617	0.0436	0.431	0.0870	0.211	0.00727	0.0598
70	0.254	0.295	0.392	0.00614	0.0450	0.407	0.1019	0.234	0.00810	0.0720
80	0.249	0.301	0.387	0.00636	0.0455	0.386	0.1016	0.241	0.00768	0.0689
90	0.241	0.309	0.387	0.00641	0.0464	0.370	0.1102	0.259	0.00836	0.0769
100	0.239	0.306	0.388	0.00655	0.0449	0.350	0.0994	0.245	0.00656	0.0606
120	0.223	0.299	0.401	0.00594	0.0424	0.330	0.1205	0.262	0.00897	0.0732
140	0.221	0.318	0.399	0.00658	0.0471	0.309	0.1157	0.263	0.00808	0.0669
160	0.214	0.321	0.405	0.00652	0.0476	0.292	0.1189	0.262	0.00782	0.0663
180	0.207	0.315	0.405	0.00634	0.0445	0.280	0.1182	0.278	0.00797	0.0664
200	0.202	0.316	0.413	0.00616	0.0448	0.270	0.1227	0.282	0.00849	0.0680
250	0.197	0.320	0.414	0.00641	0.0454	0.244	0.1229	0.287	0.00769	0.0648
300	0.190	0.325	0.418	0.00640	0.0461	0.229	0.1303	0.287	0.00850	0.0673
350	0.187	0.324	0.417	0.00639	0.0445	0.216	0.1298	0.294	0.00813	0.0667
400	0.178	0.319	0.430	0.00598	0.0439	0.203	0.1315	0.301	0.00797	0.0657
500	0.176	0.322	0.426	0.00631	0.0437	0.188	0.1373	0.313	0.00850	0.0704

Table S3: The fit parameters for fixed loop or tail lengths.

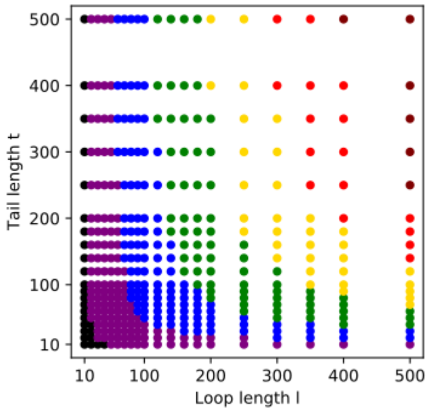

Figure S5: Domination number d as a function of tail lenght t and loop length l.

S3 Shape parameters of lasso loop

S3.1 Mean radius of gyration, distension, asphericity, and prolatness

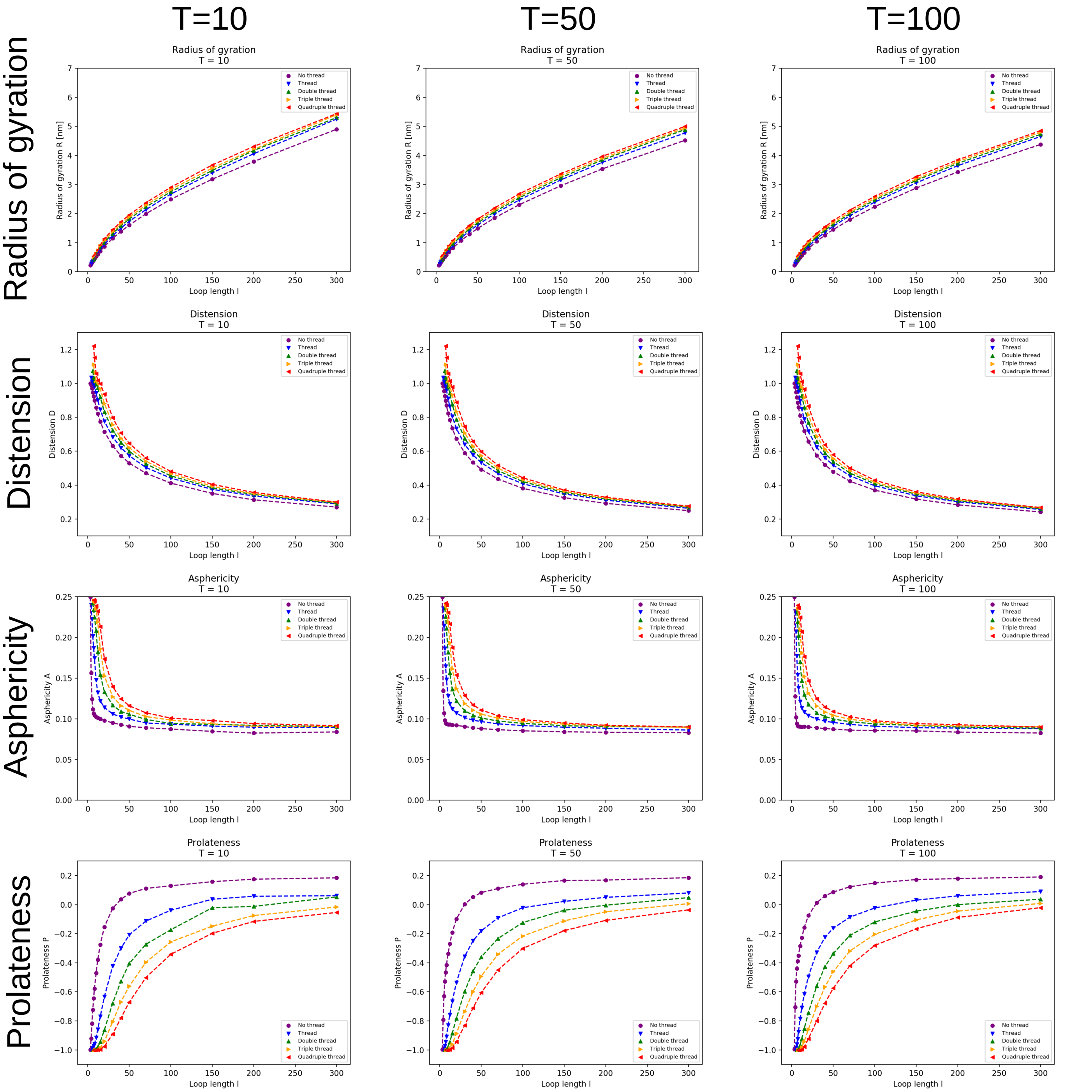

Figure S6: The temperature dependence of radius of gyration, distension, asphericity, and prolatness. The temperatures are given in Gromacs reduced units.

Radius of gyration

**Thread thickness**	a_R	**Error**	ν	**Error**	c_R	**Error**
**0**	0.160175	± 0.003201	0.585258	± 0.003067	-0.130364	± 0.01284
**1**	0.163818	± 0.003623	0.589809	± 0.0034	-0.0825764	± 0.01477
**2**	0.175243	± 0.002081	0.580079	± 0.001818	-0.0645895	± 0.008199
**3**	0.163822	± 0.005273	0.591032	± 0.00495	0.0310455	± 0.02158
**4**	0.179506	± 0.001129	0.576588	± 0.0009622	0.0401297	± 0.004397

Radius of gyration with fixed $\nu = 0.59$

**Thread thickness**	a_R	**Error**	c_R	**Error**
**0**	0.155319	± 0.0001625	-0.111104	± 0.002779
**1**	0.163615	± 0.0001562	-0.0817651	± 0.002671
**2**	0.164301	± 0.0002207	-0.0215718	± 0.0.003774
**3**	0.164923	± 0.0002301	0.0266388	± 0.003935
**4**	0.164513	± 0.0002798	0.0987288	± 0.004785

Asphericity

**Thread thickness**	a_A	**Error**	μ	**Error**	A_∞	**Error**
**0**	—	—	—	—	0.0818678	—
**1**	0.169118	± 0.04401	-0.710574	± 0.09109	0.0847535	± 0.0009532
**2**	0.316019	± 0.04399	-0.778194	± 0.04714	0.0849596	± 0.0009532
**3**	0.85873	± 0.1704	-0.999367	± 0.06287	0.0870523	± 0.0007168
**4**	1.2737	± 1.2737	-1.03661	± 0.04096	0.0869729	± 0.0005741

Prolateness

**Thread thickness**	a_P	**Error**	P_∞	**Error**
**0**	-1.32633	± 0.07353	0.293166	± 0.01005
**1**	-3.11353	± 0.1178	0.322087	± 0.01611
**2**	-4.53756	± 0.1688	0.379018	± 0.02309
**3**	-5.42652	± 0.1116	0.402923	± 0.01526
**4**	-6.0385	± 0.1089	0.1089	± 0.01489

Table S4: The fitting errors for parameters describing the radius of gyration, asphericity and prolateness.

S4 Protein data

S4.1 Scaling of the area of minimal surface in proteins.

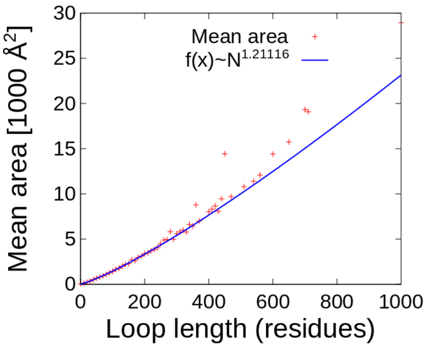

Figure S7: The mean area of the minimal surface spanned on the protein covalent loop with the best fit. The data come from LassoProt database [2].

The areas were binned with the bin width 10 residues. For each bin the mean surface was calculated. The mean surface was fitted with the following formula:

$$f(x) = S_0 + A \cdot N^{\mu} \quad (3)$$

Due to poor statistics for large loops, the fit was done only for loops with at most 250 residues. The obtained parameters:

Parameter	Value	Fiting assymptotic error
μ	1.21116	0.03081 (2.544%)
A	5.37387	0.9314 (17.33%)
S_0	37.1597	38.2 (102.8%)

Table S5: The obtained fitting parameters for the mean area scalling as a function of protein loop length.

With the high error of S_0 parameter not influencing the general consideration about the scalling of the surface discussed in main text.

S4.2 Calculation of the expected number of non-trivial proteins

The calculation of the expected number of the non-trivial proteins followed the algorithm:

1. For a given protein chain stored in PDB file:
   (a) Determine its length;
   (b) Identify all N_{loop} lasso loops (pieces of chain closed by covalent bridge);
   (c) Determine all lengths of lasso loops and corresponding lasso tails;
   (d) Estimate the piercing probability $P(A_{i,j})$ for each lasso loop $i \in \{1, ..., N_{loop}\}$ and for each lasso tail $j \in \{N, C\}$ separately;
   (e) Calculate the probability $P_{PDB} = P(A_{1,N} \cup A_{1,C} \cup ... \cup A_{N_{loop},N} \cup A_{N_{loop},C})$ of at least one piercing in the chain, using the inclusion-exclusion principle;
2. Group all the lasso loops with the same sequential length into one set. If the sets for consecutive lasso loop lengths are smaller than 20 representants, merge them. In particular, as there are only singular cases of structures with over 900 residues in the loop, merge them all into one set.
3. For each set consisting of N_{chain} chains, calculate the probability distribution of obtaining exactly k chains with at least one threading $P(k)$:
   (a) Generate all $\binom{N_{chain}}{k}$ k-element subsets of the set of chains;
   (b) Calculate the product of the threading probabilities P_{PDB} of the chains in the k-element subset multiplied by the product of the non-threading probabilites $P_{N;PDB}$ of the $N_{chain} - k$ other chains ($P_{N;PDB} = 1 - P_{PDB}$);
   (c) Sum the probabilities corresponding to all subsetss.
4. Calculate the expected number of threaded lasso loops $\mathbb{E}P = \sum_{k=0}^{\infty} k \cdot P(k)$ for each set;
5. Round the expected number to the neares integer and present it as a function of the chain length.

S4.3 Non-trivial lassos

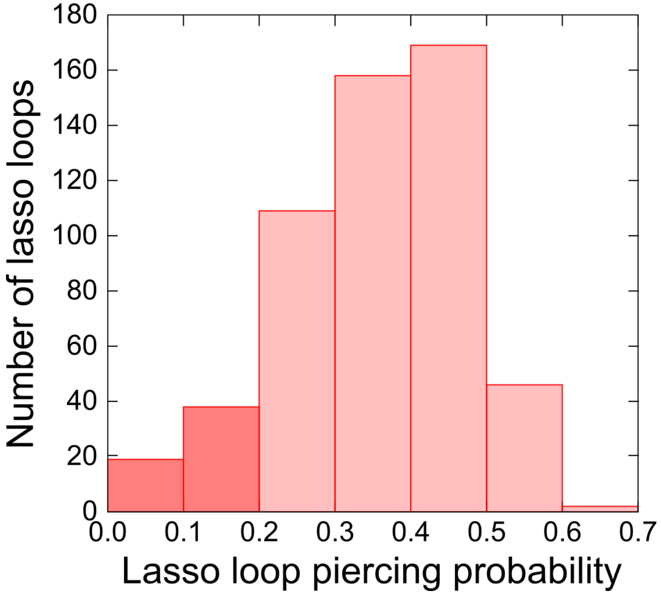

Figure S8: The spectrum of threading probabilities. The plot shows the histogram of threading probabilities calculated for each lasso loop (portion of the chain closed by covalent bridge) in the analyzed set of proteins. The darker red bars encompass the structures used in the analysis of the lasso motif function.

Table S6: The table with structural parameters of analyzed non-trivial lassos. Open and close are the indices of the bridge, tail1 and tail2 are the tail lengths, a, b, c are the ellipsoid semi-axis lengths, Asph and Pred_A are calculated and predicted asphericity, Prolat and Pred_P are the calculated and predicted prolatness, motif_P is the motif probability, "Organism" is the organism of origin derived from PDB "ORGANISM SCIENTIFIC" line, "Function" is the function derived from PDB HEADER line. The notation in type denotes the number, direction and piercing tail as in [2]. The blue structures are the miniproteins (lasso peptides) with amide-bridge-closed loop. The horizontal line separates the structures with threading probability < 0.2.

code	chain	type	open	close	tail1	tail2	a	b	c	Asph	Pred_A	Prolat	Pred_P	motif_P	Organism	Function
5JPL	A	L-1C	1	8	0	0	0.326	0.367	0.466	0.012	0.152	0.656	-1.000	0.005	NOCARDIOPSIS ALBA	ANTIBIOTIC
5XM4	A	L-1C	1	8	0	0	0.340	0.347	0.452	0.009	0.152	0.985	-1.000	0.005	NOVOSPHINGOBIUM SUBTERRANEUM	UNKNOWN FUNCTION
4NAG	A	L-1C	1	7	0	0	0.279	0.362	0.447	0.018	0.174	0.026	-1.000	-0.007	XANTHOMONAS GARDNERI	BIOSYNTHETIC PROTEIN
2N5C	A	L-1C	1	8	0	0	0.341	0.367	0.491	0.013	0.152	0.882	-1.000	-0.011	STREPTOMYCES LEEUWENHOEKII	CELL INVASION
5GVO	A	L-1C	1	9	0	0	0.365	0.377	0.475	0.007	0.138	0.958	-1.000	0.017	PLANOMONOSPORA SPHAERICA	UNKNOWN FUNCTION
2LX6	A	L-1C	1	8	0	0	0.316	0.411	0.501	0.017	0.152	-0.053	-1.000	0.020	CAULOBACTER SEGNIS	UNKNOWN FUNCTION
6B5W	A	L-1C	1	8	0	0	0.330	0.359	0.476	0.013	0.152	0.837	-1.000	0.020	ASTICCACAULIS BENEVESTITUS	UNKNOWN FUNCTION
2MFV	A	L-1C	1	7	0	0	0.301	0.358	0.457	0.015	0.174	0.453	-1.000	-0.024	XANTHOMONAS GARDNERI	UNKNOWN FUNCTION
2M37	A	L-1C	1	9	0	0	0.354	0.389	0.504	0.012	0.138	0.786	-1.000	0.025	ASTICCACAULIS EXCENTRICUS	UNKNOWN FUNCTION
3NJW	A	L-1C	1	9	0	0	0.349	0.387	0.493	0.011	0.138	0.717	-1.000	0.025	STREPTOMYCES SP.	ANTIMICROBIAL PROTEIN
5D9E	A	L-1C	1	9	0	0	0.367	0.408	0.534	0.013	0.138	0.752	-1.000	0.025	CAULOBACTER SEGNIS	UNKNOWN FUNCTION

Continued on next page

Table S6 – *Continued from previous page*

code	chain	type	open	close	tail1	tail2	a	b	c	Asph	Pred_A	Prolat	Pred_P	motif_P	org	func
2LS1	A	L-1C	1	9	0	0	0.373	0.381	0.523	0.013	0.138	0.990	-1.000	0.032	STREPTOMYCES SVICEUS	ANTIMICROBIAL PROTEIN
1RPB	A	L-1C	1	9	0	0	0.378	0.408	0.543	0.013	0.138	0.866	-1.000	0.039	ACTINOMYCETE SP9440	REPLICATION INHIBITOR
2MW3	A	L-1C	1	9	0	0	0.350	0.381	0.498	0.012	0.138	0.832	-1.000	0.039	STREPTOMONOSPORA ALBA	UNKNOWN FUNCTION
5JQF	A	L-1C	1	9	0	0	0.354	0.373	0.483	0.010	0.138	0.916	-1.000	0.039	SPHINGOPYXIS ALASKENSIS RB2256	UNKNOWN FUNCTION
5ZCN	A	L-1C	1	9	0	0	0.349	0.405	0.506	0.012	0.138	0.467	-1.000	0.039	BREVUNDIMONAS DIMINUTA	UNKNOWN FUNCTION
2LTI	A	L-1C	1	9	0	0	0.348	0.424	0.539	0.016	0.138	0.334	-1.000	0.051	ASTICCACAULIS EXCENTRICUS	ANTIMICROBIAL PROTEIN
2M8F	A	L-1C	1	9	0	0	0.340	0.431	0.534	0.017	0.138	0.117	-1.000	0.057	ASTICCACAULIS EXCENTRICUS	UNKNOWN FUNCTION
1IJV	A	L-1C	12	27	11	11	0.450	0.592	0.696	0.015	0.105	-0.263	-0.715	0.088	HOMO SAPIENS	DEFENSIN
3QTE	A	L-1C	6	20	5	5	0.415	0.589	0.676	0.019	0.107	-0.536	-0.750	0.101	HOMO SAPIENS	ANTIMICROBIAL PROTEIN
1FD3	A	L-1C	15	30	14	14	0.458	0.585	0.688	0.013	0.105	-0.179	-0.715	0.103	HOMO SAPIENS	ANTIMICROBIAL PROTEIN
1TV0	A	L-1C	6	21	5	5	0.452	0.584	0.670	0.012	0.105	-0.350	-0.715	0.103	MUS MUSCULUS	ANTIMICROBIAL PROTEIN
1UT3	A	L-1C	12	27	11	11	0.460	0.553	0.650	0.010	0.105	0.027	-0.715	0.103	APTENODYTES PATAGONICUS	ANTIBIOTIC
4LB1	A	L-1C	4	19	3	3	0.460	0.582	0.683	0.013	0.105	-0.164	-0.715	0.103	HOMO SAPIENS	ANTIMICROBIAL PROTEIN
1B8W	A	L-1C	16	32	15	15	0.438	0.629	0.698	0.017	0.104	-0.709	-0.684	0.105	ORNITHORHYNCHUS ANATINUS	TOXIN
1D6B	A	L-1C	16	32	15	15	0.433	0.674	0.749	0.024	0.104	-0.772	-0.684	0.105	ORNITHORHYNCHUS ANATINUS	TOXIN
2JR3	A	L-1C	16	32	15	15	0.494	0.594	0.733	0.013	0.104	0.280	-0.684	0.105	PELODISCUS SINENSIS	ANTIMICROBIAL PROTEIN
1EWS	A	L-1C	5	19	4	4	0.390	0.609	0.678	0.024	0.107	-0.771	-0.750	0.107	ORYCTOLAGUS CUNICULUS	ANTIMICROBIAL PROTEIN
1KJ6	A	L-1C	18	33	17	17	0.480	0.546	0.666	0.009	0.105	0.484	-0.715	0.110	HOMO SAPIENS	ANTIBIOTIC
1ZMM	A	L-1C	4	19	3	3	0.456	0.589	0.692	0.014	0.105	-0.221	-0.715	0.110	HOMO SAPIENS	ANTIMICROBIAL PROTEIN
2RNG	A	L-1C	52	70	51	51	0.440	0.682	0.776	0.025	0.101	-0.679	-0.629	0.113	TACHYPLEUS TRIDENTATUS	ANTIMICROBIAL PROTEIN
2MN3	A	L-1C	16	30	15	15	0.410	0.615	0.674	0.020	0.107	-0.802	-0.750	0.113	ORNITHORHYNCHUS ANATINUS	ANTIMICROBIAL PROTEIN
5KI9	A	L-1C	15	29	14	14	0.422	0.585	0.679	0.018	0.107	-0.442	-0.750	0.113	HOMO SAPIENS	ANTIMICROBIAL PROTEIN
2LG6	A	L-1C	8	23	7	7	0.440	0.551	0.644	0.012	0.105	-0.149	-0.715	0.116	GALLUS GALLUS	ANTIMICROBIAL PROTEIN
2B5B	A	L-1C	8	29	7	7	0.490	0.539	0.609	0.004	0.099	0.295	-0.562	0.118	CARETTA CARETTA	ANTIBIOTIC
2JTO	A	L-1C	47	64	46	46	0.436	0.642	0.719	0.020	0.103	-0.694	-0.655	0.120	RHIPICEPHALUS BURSA	HYDROLASE INHIBITOR
2K2Z	A	L-1C	47	64	10	10	0.434	0.664	0.723	0.021	0.103	-0.834	-0.655	0.120	RHIPICEPHALUS BURSA	HYDROLASE INHIBITOR
4X0N	B	L-1C	1016	1033	15	15	0.404	0.744	0.789	0.035	0.103	-0.949	-0.655	0.120	SUS SCROFA	HYDROLASE/HYDROLASE INHIBITOR
5LAH	A	L-1C	17	35	16	16	0.457	0.662	0.740	0.019	0.101	-0.688	-0.629	0.121	URTICINA EQUES	TOXIN
5LCS	A	L-1C	18	33	17	17	0.444	0.563	0.648	0.011	0.105	-0.273	-0.715	0.122	GALLUS GALLUS	IMMUNE SYSTEM
2MJK	A	L-1C	12	28	11	11	0.391	0.681	0.742	0.032	0.104	-0.882	-0.684	0.125	GALLUS GALLUS	ANTIMICROBIAL PROTEIN
2K2Y	A	L-1C	10	27	9	9	0.495	0.614	0.737	0.013	0.103	0.024	-0.655	0.127	RHIPICEPHALUS BURSA	HYDROLASE INHIBITOR
2MXQ	A	L-1C	4	20	3	3	0.523	0.610	0.719	0.008	0.104	0.192	-0.684	0.131	EQUUS CABALLUS	ANTIMICROBIAL PROTEIN
5VG2	A	L-6C	56	99	0	0	0.887	1.293	1.373	0.016	0.117	-0.895	-0.699	0.133	TETRANYCHUS URTICAE	OXIDOREDUCTASE
6BDJ	A	L-6C	56	99	0	0	0.886	1.289	1.367	0.016	0.117	-0.898	-0.699	0.133	TETRANYCHUS URTICAE	METAL BINDING PROTEIN
4QI7	A	L+6N	167	211	165	165	1.100	1.374	1.723	0.017	0.117	0.206	-0.689	0.134	NEUROSPORA CRASSA	OXIDOREDUCTASE
2B5B	A	L-1C	4	30	3	3	0.507	0.613	0.653	0.005	0.097	-0.695	-0.476	0.140	CARETTA CARETTA	ANTIBIOTIC
4GV5	A	L-1C	11	30	10	10	0.493	0.635	0.744	0.014	0.101	-0.219	-0.605	0.142	CROTALUS DURISSUS TERRIFICUS	TOXIN
1UDK	A	L-1C	20	41	19	19	0.461	0.739	0.765	0.022	0.099	-0.973	-0.562	0.142	NAJA NIGRICOLLIS	UNKNOWN FUNCTION
3OZP	A	L-1N	36	55	13	13	0.476	0.741	0.812	0.023	0.101	-0.819	-0.605	0.148	OSTRINIA FURNACALIS	HYDROLASE/HYDROLASE INHIBITOR
5JBT	Y	L-1N	30	51	12	12	0.476	0.781	0.860	0.028	0.099	-0.832	-0.562	0.156	HOMO SAPIENS	HYDROLASE/HYDROLASE INHIBITOR
3FP7	J	L-1N	30	51	14	14	0.496	0.731	0.787	0.018	0.099	-0.855	-0.562	0.169	RATTUS NORVEGICUS	HYDROLASE/HYDROLASE INHIBITOR
5NX3	D	L-1N	30	51	14	14	0.497	0.740	0.780	0.017	0.099	-0.924	-0.562	0.169	HOMO SAPIENS	HYDROLASE
1WQK	A	L-1C	6	30	5	5	0.586	0.737	0.842	0.011	0.097	-0.306	-0.507	0.176	ANTHOPLEURA ELEGANTISSIMA	TOXIN
1BDS	A	L-1C	6	32	5	5	0.592	0.817	0.879	0.013	0.097	-0.817	-0.476	0.181	ANEMONIA SULCATA	ANTI-HYPERTENSIVE
2XFD	A	L-1N	90	101	88	88	0.438	0.470	0.626	0.013	0.117	0.891	-0.880	0.192	ESCHERICHIA COLI	SUGAR BINDING PROTEIN
3NGG	A	L-1C	10	35	5	5	0.556	0.790	0.901	0.018	0.097	-0.569	-0.491	0.199	OXYURANUS MICROLEPIDOTUS	ANTIBIOTIC
2UUX	A	L-1N	52	69	31	31	0.447	0.670	0.715	0.018	0.103	-0.891	-0.655	0.200	RHIPICEPHALUS APPENDICULATUS	INHIBITOR
2Q8T	A	L-2C	16	40	6	6	0.603	0.942	1.085	0.027	0.112	-0.639	-0.759	0.200	HOMO SAPIENS	CYTOKINE
2IKE	A	L-1C	83	113	12	12	0.617	0.708	0.888	0.012	0.096	0.537	-0.423	0.202	MANDUCA SEXTA	HYDROLASE
5UIW	B	L-2C	10	34	10	10	0.601	0.945	1.087	0.027	0.112	-0.648	-0.759	0.203	HOMO SAPIENS	SIGNALING PROTEIN
5LTL	A	L-2C	14	37	6	6	0.607	0.878	1.023	0.021	0.113	-0.494	-0.782	0.204	HOMO SAPIENS	CYTOKINE
4RWS	C	L-2C	11	35	10	10	0.608	0.954	1.098	0.027	0.112	-0.641	-0.759	0.206	HOMO SAPIENS	SIGNALING PROTEIN, HYDROLASE

Continued on next page

Table S6 – *Continued from previous page*

code	chain	type	open	close	tail1	tail2	a	b	c	Asph	Pred_A	Prolat	Pred_P	motif_P	org	func
1CM9	A	L-2C	14	38	7	7	0.610	0.935	1.077	0.025	0.112	-0.619	-0.759	0.207	HUMAN HERPESVIRUS 8	CHEMOKINE
2HCC	A	L-2C	6	30	5	5	0.621	0.898	1.053	0.022	0.112	-0.467	-0.759	0.207	HOMO SAPIENS	CHEMOKINE
1DTV	A	L-1N	19	43	18	18	0.629	0.765	0.885	0.009	0.097	-0.110	-0.507	0.208	HIRUDO MEDICINALIS	HYDROLASE INHIBITOR
2LVX	A	L+1C	408	437	51	51	0.455	1.165	1.180	0.066	0.096	-0.999	-0.435	0.210	SCHIZOSACCHAROMYCES POMBE	HYDROLASE, SUGAR BINDING PROTEIN
1M8A	A	L-2C	6	32	1	1	0.595	0.993	1.117	0.030	0.111	-0.770	-0.718	0.212	HOMO SAPIENS	CYTOKINE
2IKD	A	L-1C	23	54	12	12	0.655	0.688	0.899	0.010	0.095	0.931	-0.412	0.213	MANDUCA SEXTA	HYDROLASE
4HCS	A	L-2C	15	40	3	3	0.590	0.967	1.074	0.028	0.111	-0.805	-0.738	0.215	DANIO RERIO	SIGNALING PROTEIN
2R3Z	A	L-2C	9	36	8	8	0.626	1.030	1.129	0.028	0.110	-0.848	-0.699	0.215	MUS MUSCULUS	ATTRACTANT
1F9P	A	L-2C	25	51	19	19	0.581	1.035	1.133	0.034	0.111	-0.875	-0.718	0.215	HOMO SAPIENS	BLOOD CLOTTING
4XT3	B	L-2C	8	34	7	7	0.617	0.989	1.109	0.027	0.111	-0.761	-0.718	0.215	CYTOMEGALOVIRUS	VIRAL PROTEIN/SIGNALLING PROTEIN
1SHI	A	L-1C	5	33	4	4	0.516	0.995	1.037	0.039	0.096	-0.977	-0.448	0.216	STICHODACTYLA HELIANTHUS	NEUROTOXIN
2L4N	A	L-2C	8	34	7	7	0.592	0.985	1.108	0.030	0.111	-0.772	-0.718	0.217	HOMO SAPIENS	CYTOKINE
1BIK	A	L-1N	51	72	26	26	0.500	0.745	0.793	0.018	0.099	-0.896	-0.562	0.217	HOMO SAPIENS	GLYCOPROTEIN
6FWN	A	L-1C	2528	2570	35	35	0.551	1.629	1.671	0.082	0.094	-0.995	-0.312	0.217	HOMO SAPIENS	BLOOD CLOTTING
5M0W	A	L-1N	60	78	38	38	0.484	0.686	0.775	0.018	0.101	-0.616	-0.629	0.220	MUS MUSCULUS	SIGNALING PROTEIN
5C67	C	L-1N	30	51	27	27	0.490	0.757	0.799	0.020	0.099	-0.927	-0.562	0.220	HOMO SAPIENS	HYRDOLASE/HYDROLASE INHIBITOR
2MGS	A	L-2C	13	39	5	5	0.594	1.036	1.144	0.033	0.111	-0.847	-0.718	0.220	HOMO SAPIENS	SIGNALING PROTEIN
1UDK	A	L-1C	7	37	6	6	0.680	0.891	1.023	0.013	0.096	-0.382	-0.423	0.221	NAJA NIGRICOLLIS	UNKNOWN FUNCTION
1RJT	A	L-2C	9	36	8	8	0.582	0.981	1.105	0.031	0.110	-0.775	-0.699	0.222	HOMO SAPIENS	CYTOKINE
4BD9	B	L-1N	29	50	28	28	0.492	0.760	0.806	0.020	0.099	-0.916	-0.562	0.223	HOMO SAPIENS	HYDROLASE/HYDROLASE INHIBITOR
2W8X	A	L-1N	51	69	40	40	0.439	0.632	0.677	0.016	0.101	-0.859	-0.629	0.223	RHIPICEPHALUS APPENDICULATUS	MEMBRANE PROTEIN
2VGA	A	L-3C	112	152	106	106	1.013	1.360	1.666	0.020	0.111	-0.109	-0.647	0.223	VACCINIA VIRUS	VIRAL PROTEIN
2JTO	A	L-1C	10	27	9	9	0.458	0.598	0.708	0.015	0.103	-0.209	-0.655	0.224	RHIPICEPHALUS BURSA	HYDROLASE INHIBITOR
2MP1	A	L-2C	8	34	7	7	0.616	0.903	1.028	0.021	0.111	-0.619	-0.718	0.224	HOMO SAPIENS	SIGNALING PROTEIN
5EKI	A	L-2C	8	34	2	2	0.588	1.023	1.133	0.033	0.111	-0.837	-0.718	0.224	HOMO SAPIENS	IMMUNE SYSTEM
2GRK	A	LL+4,-3	139	178	131	131	1.095	1.301	1.639	0.014	0.120	0.405	-0.745	0.226	ECTROMELIA VIRUS	VIRAL PROTEIN
1OWT	A	L-1C	144	174	20	20	0.486	0.883	0.933	0.034	0.096	-0.952	-0.423	0.226	HOMO SAPIENS	APOPTOSIS
2JH1	A	LS3+–N	91	127	79	79	0.649	1.134	1.263	0.034	0.114	-0.824	-0.697	0.226	TOXOPLASMA GONDII	CELL ADHESION
1KSQ	A	L-1N	22	47	21	21	0.528	0.699	0.726	0.009	0.097	-0.928	-0.491	0.227	HOMO SAPIENS	PROTEIN BINDING
4ZAI	A	L-2C	11	38	11	11	0.608	1.040	1.129	0.030	0.110	-0.888	-0.699	0.227	HOMO SAPIENS	CYTOKINE
2ODY	E	L-1N	46	67	30	30	0.499	0.739	0.783	0.017	0.099	-0.909	-0.562	0.227	BOS TAURUS	BLOOD CLOTTING
2HDL	A	L-2C	3	29	2	2	0.589	0.916	1.059	0.026	0.111	-0.621	-0.718	0.229	HOMO SAPIENS	CYTOKINE
1Q25	A	L+1C	385	419	380	380	0.474	1.362	1.364	0.077	0.095	-1.000	-0.380	0.233	BOS TAURUS	PROTEIN TRANSPORT
3C6E	C	L-1C	34	68	33	33	0.528	1.048	1.076	0.041	0.095	-0.991	-0.380	0.233	DENGUE VIRUS	VIRAL PROTEIN
6EHZ	A	L-2C	9	34	8	8	0.565	0.936	1.030	0.028	0.111	-0.837	-0.738	0.234	MUS MUSCULUS	CYTOKINE
2J6D	A	L-1N	35	56	34	34	0.463	0.813	0.841	0.030	0.099	-0.980	-0.562	0.235	CONUS STRIATUS	TOXIN
1E9T	A	L-1C	11	37	10	10	0.522	0.782	0.835	0.018	0.097	-0.886	-0.476	0.236	HOMO SAPIENS	CELL MOTILITY FACTOR
2PSP	A	L-1C	58	84	57	57	0.546	0.806	0.879	0.019	0.097	-0.809	-0.476	0.236	SUS SCROFA	SIGNALING PROTEIN
2MSX	A	LS3+–C	50	92	3	3	0.788	1.020	1.091	0.009	0.110	-0.775	-0.625	0.237	HOMO SAPIENS	HYDROLASE INHIBITOR
1TAP	A	L-1N	33	55	32	32	0.490	0.813	0.888	0.028	0.098	-0.859	-0.542	0.238	ORNITHODOROS MOUBATA	PROTEINASE INHIBITOR
1YC0	I	L-1N	275	296	37	37	0.497	0.736	0.786	0.018	0.099	-0.883	-0.562	0.240	HOMO SAPIENS	HYDROLASE/INHIBITOR
1GP0	A	L+1C	1598	1634	83	83	0.493	1.348	1.372	0.073	0.095	-0.998	-0.361	0.241	HOMO SAPIENS	RECEPTOR
1ADZ	A	L-1N	39	60	38	38	0.471	0.752	0.786	0.022	0.099	-0.956	-0.562	0.242	HOMO SAPIENS	HYDROLASE
4OIE	A	L-1C	291	312	115	115	0.507	0.839	0.935	0.029	0.099	-0.796	-0.562	0.245	WEST NILE VIRUS	VIRAL PROTEIN
2UUX	A	L-1C	24	51	3	3	0.531	0.995	1.083	0.039	0.096	-0.901	-0.462	0.248	RHIPICEPHALUS APPENDICULATUS	INHIBITOR
6EPK	B	L-1C	530	565	29	29	0.525	1.082	1.108	0.044	0.095	-0.993	-0.370	0.248	YELLOW FEVER VIRUS	VIRAL PROTEIN
6CWS	A	L-2C	11	39	10	10	0.573	0.989	1.068	0.031	0.110	-0.902	-0.681	0.249	HOMO SAPIENS	CYTOKINE
2OIZ	H	L-1C	130	161	71	71	0.631	0.975	1.115	0.025	0.095	-0.653	-0.412	0.250	ALCALIGENES FAECALIS	OXIDOREDUCTASE
1C01	A	L-1C	23	49	22	22	0.641	0.825	0.966	0.013	0.097	-0.225	-0.476	0.251	MACADAMIA INTEGRIFOLIA	ANTIMICROBIAL PROTEIN
1HI7	A	L-1C	7	33	6	6	0.521	0.790	0.842	0.019	0.097	-0.896	-0.476	0.251	HOMO SAPIENS	GROWTH FACTOR
2M05	A	L-1C	146	182	13	13	0.524	1.088	1.097	0.044	0.095	-0.999	-0.361	0.252	CAENORHABDITIS ELEGANS	UNKNOWN FUNCTION
3AGN	A	L-3C	1	54	0	0	0.886	1.075	1.215	0.008	0.106	-0.250	-0.526	0.253	USTILAGO SPHAEROGENA	HYDROLASE

Continued on next page

Table S6 – *Continued from previous page*

code	chain	type	open	close	tail1	tail2	a	b	c	Asph	Pred_A	Prolat	Pred_P	motif_P	org	func
4BQD	A	L-1N	51	72	49	49	0.501	0.748	0.790	0.018	0.099	-0.920	-0.562	0.255	HOMO SAPIENS	BLOOD CLOTTING
2K35	A	L-1C	5	48	4	4	0.709	0.790	0.870	0.003	0.094	0.004	-0.305	0.259	HYDRA	ANTIMICROBIAL PROTEIN
2JH1	A	LS3+–N	181	226	169	169	0.662	1.424	1.519	0.051	0.109	-0.955	-0.595	0.262	TOXOPLASMA GONDII	CELL ADHESION
1OH1	A	L+1N	16	55	15	15	0.710	0.987	1.134	0.017	0.094	-0.502	-0.335	0.264	STAPHYLOCOCCUS AUREUS	CYSTEINE PROTEINASE INHIBITOR
2OIZ	H	L-2C	81	113	22	22	0.641	0.876	0.970	0.014	0.108	-0.666	-0.618	0.264	ALCALIGENES FAECALIS	OXIDOREDUCTASE
4XHJ	B	L-2C	49	80	20	20	0.648	1.112	1.246	0.033	0.108	-0.799	-0.633	0.265	HUMAN HERPESVIRUS	VIRAL PROTEIN
6MEI	C	L-2C	459	486	49	49	0.809	0.904	1.143	0.011	0.110	0.669	-0.699	0.267	HEPACIVIRUS C	IMMUNE SYSTEM
5ZE3	A	L-3C	573	625	251	251	0.824	1.497	1.637	0.036	0.106	-0.885	-0.534	0.269	HOMO SAPIENS	OXIDOREDUCTASE
4F2M	E	L-1C	594	621	87	87	0.558	0.983	1.033	0.031	0.096	-0.958	-0.462	0.270	MUS MUSCULUS	VIRAL PROTEIN
1C01	A	L-1N	11	64	10	10	0.808	0.883	1.004	0.004	0.093	0.398	-0.245	0.272	MACADAMIA INTEGRIFOLIA	ANTIMICROBIAL PROTEIN
5D71	A	L-2N	88	121	79	79	0.759	0.990	1.220	0.018	0.107	-0.003	-0.604	0.272	HOMO SAPIENS	IMMUNE SYSTEM
3HZB	A	L-1N	9	70	8	8	0.838	0.907	0.980	0.002	0.093	0.033	-0.208	0.273	FLAVOBACTERIUM JOHNSONIAE	METAL BINDING PROTEIN
4BD9	B	L-1N	81	102	80	80	0.494	0.744	0.787	0.018	0.099	-0.918	-0.562	0.273	HOMO SAPIENS	HYDROLASE/HYDROLASE INHIBITOR
2GMF	A	L-2N	88	121	84	84	0.770	0.994	1.230	0.018	0.107	0.043	-0.604	0.274	HOMO SAPIENS	GROWTH FACTOR
6ATK	E	L-1C	369	396	71	71	0.587	0.882	0.923	0.018	0.096	-0.944	-0.462	0.274	HOMO SAPIENS	HYDROLASE/VIRAL PROTEIN
1BIK	A	L-1N	107	128	82	82	0.496	0.760	0.810	0.020	0.099	-0.900	-0.562	0.274	HOMO SAPIENS	GLYCOPROTEIN
2Y1B	A	L-1C	74	118	27	27	0.619	1.169	1.195	0.036	0.094	-0.993	-0.298	0.274	ESCHERICHIA COLI	MEMBRANE PROTEIN
3KTM	A	L-1C	144	174	116	116	0.488	0.912	0.953	0.036	0.096	-0.972	-0.423	0.275	HOMO SAPIENS	CELL ADHESION, SIGNALING PROTEIN
5HQN	A	L-1N	225	248	58	58	0.600	0.669	0.775	0.006	0.098	0.347	-0.524	0.275	MUS MUSCULUS	HYDROLASE
6F99	A	L+1C	193	227	105	105	0.466	1.414	1.421	0.083	0.095	-1.000	-0.380	0.275	SACCHAROMYCES CEREVISIAE	SUGAR BINDING PROTEIN
1PNB	B	L-1C	15	62	14	14	0.600	1.083	1.094	0.031	0.094	-0.998	-0.279	0.277	BRASSICA NAPUS	SEED STORAGE PROTEIN
4KYP	A	L-1N	43	69	42	42	0.600	0.808	0.842	0.010	0.097	-0.928	-0.476	0.278	HOTTENTOTTA JUDAICUS	TOXIN
1DTV	A	L-1N	22	58	21	21	0.723	0.859	0.958	0.006	0.095	-0.272	-0.361	0.279	HIRUDO MEDICINALIS	HYDROLASE INHIBITOR
2ODY	E	L-1N	114	135	98	98	0.497	0.748	0.789	0.018	0.099	-0.925	-0.562	0.280	BOS TAURUS	BLOOD CLOTTING
2KER	A	L-1N	43	70	42	42	0.558	0.945	1.017	0.029	0.096	-0.905	-0.462	0.284	STREPTOMYCES PARVULUS	HYDROLASE INHIBITOR
1WKT	A	L-1C	27	58	26	26	0.598	1.038	1.122	0.031	0.095	-0.901	-0.412	0.284	WILLIOPSIS SATURNUS VAR. MRAKII	TOXIN
2ZOT	C	LS3–+C	44	128	0	0	1.088	1.138	1.239	0.001	0.100	0.544	-0.359	0.284	HOMO SAPIENS	CELL ADHESION
2ZOU	B	LS3–+C	44	128	1	1	1.093	1.114	1.219	0.001	0.100	0.898	-0.359	0.284	HOMO SAPIENS	CELL ADHESION
3B4V	D	L-1N	22	66	16	16	0.636	0.896	0.971	0.015	0.094	-0.801	-0.298	0.285	HOMO SAPIENS	HORMONE REGULATOR COMPLEX
2W86	A	L-1N	58	83	57	57	0.554	0.670	0.678	0.004	0.097	-0.984	-0.491	0.286	HOMO SAPIENS	GLYCOPROTEIN
1HX2	A	L-1N	21	60	20	20	0.606	0.819	0.864	0.011	0.094	-0.882	-0.335	0.287	BOMBINA BOMBINA	HYDROLASE INHIBITOR
2EHG	A	L+3N	58	145	57	57	1.002	1.224	1.232	0.004	0.099	-0.995	-0.348	0.287	SULFOLOBUS TOKODAII	HYDROLASE
4RUN	A	L-3N	59	151	51	51	1.008	1.195	1.238	0.004	0.099	-0.862	-0.330	0.288	HOMO SAPIENS	TRANSPORT PROTEIN
2JOP	A	L-1C	60	125	35	35	0.816	1.053	1.083	0.007	0.093	-0.953	-0.192	0.288	HOMO SAPIENS	IMMUNE SYSTEM
1WKT	A	L-1N	11	72	10	10	0.819	0.994	1.114	0.008	0.093	-0.312	-0.208	0.289	WILLIOPSIS SATURNUS VAR. MRAKII	TOXIN
4ODD	A	L-3N	62	154	53	53	1.000	1.193	1.218	0.004	0.099	-0.951	-0.330	0.289	CANIS LUPUS FAMILIARIS	ALLERGEN
1DTV	A	L-1N	18	62	17	17	0.685	0.809	0.889	0.006	0.094	-0.358	-0.298	0.290	HIRUDO MEDICINALIS	HYDROLASE INHIBITOR
4BD9	B	L-1N	140	161	139	139	0.496	0.743	0.788	0.018	0.099	-0.909	-0.562	0.291	HOMO SAPIENS	HYDROLASE/HYDROLASE INHIBITOR
2YG2	B	L-3N	95	183	66	66	1.007	1.203	1.303	0.005	0.099	-0.533	-0.344	0.291	HOMO SAPIENS	LIPID TRANSPORT
1OK0	A	L-1N	45	73	44	44	0.571	0.952	1.007	0.026	0.096	-0.940	-0.448	0.291	STREPTOMYCES TENDAE	INHIBITOR
1EPA	A	L-3N	60	154	57	57	0.993	1.232	1.252	0.005	0.098	-0.977	-0.323	0.292	RATTUS NORVEGICUS	RETINOIC ACID-BINDING PROTEIN
4PMK	B	L+1C	95	120	47	47	0.530	0.865	0.935	0.026	0.097	-0.884	-0.491	0.293	ACTINIDIA CHINENSIS	PLANT PROTEIN
6FPG	E	L+1C	104	129	63	63	0.524	0.886	0.950	0.028	0.097	-0.914	-0.491	0.294	USTILAGO MAYDIS	CELL INVASION
4CK4	A	L-3N	66	160	63	63	0.986	1.182	1.219	0.004	0.098	-0.900	-0.323	0.295	OVIS ARIES	TRANSPORT PROTEIN
1ETE	A	L-2N	93	132	92	92	0.824	1.016	1.276	0.016	0.105	0.260	-0.531	0.295	HOMO SAPIENS	CYTOKINE
2OYA	A	L-1C	446	507	29	29	0.741	0.939	1.010	0.008	0.093	-0.718	-0.208	0.296	MUS MUSCULUS	LIGAND BINDING PROTEIN
4ES7	A	L-3N	65	162	63	63	1.012	1.257	1.300	0.006	0.098	-0.915	-0.314	0.296	HOMO SAPIENS	IMMUNE SYSTEM
3KQ0	A	L-3N	72	165	71	71	1.072	1.145	1.273	0.003	0.099	0.458	-0.327	0.297	HOMO SAPIENS	SIGNALING PROTEIN
6N29	A	L-1N	792	827	28	28	0.675	0.912	1.019	0.014	0.095	-0.597	-0.370	0.297	HOMO SAPIENS	BLOOD CLOTTING
2MM2	A	L-1N	18	65	17	17	0.667	0.939	0.983	0.013	0.094	-0.925	-0.279	0.299	PYRENOPHORA TRITICI-REPENTIS	PLANT PROTEIN
5KEM	A	L+1N	121	147	68	68	0.756	0.961	1.196	0.017	0.097	0.117	-0.476	0.299	HOMO SAPIENS	VIRAL PROTEIN
4PSC	A	L-2C	55	91	24	24	0.636	1.102	1.219	0.033	0.106	-0.840	-0.566	0.300	TRICHODERMA REESEI	HYDROLASE

Continued on next page

Table S6 – *Continued from previous page*

code	chain	type	open	close	tail1	tail2	a	b	c	Asph	Pred_A	Prolat	Pred_P	motif_P	org	func
5MRV	A	L+1C	157	179	134	134	0.502	0.726	0.817	0.019	0.098	-0.660	-0.542	0.300	HOMO SAPIENS	HYDROLASE
4KG7	A	LS3++-C	54	123	28	28	1.001	1.264	1.499	0.013	0.102	-0.096	-0.426	0.303	MYCOBACTERIUM SMEGMATIS	HYDROLASE
1KT6	A	L-3N	70	174	69	69	1.127	1.197	1.374	0.004	0.097	0.675	-0.293	0.303	BOS TAURUS	TRANSPORT PROTEIN
3FMZ	A	L-3N	70	174	69	69	1.126	1.182	1.356	0.003	0.097	0.764	-0.293	0.303	HOMO SAPIENS	TRANSPORT PROTEIN
4H14	A	L-3N	172	252	157	157	1.111	1.253	1.493	0.008	0.100	0.426	-0.375	0.303	BOVINE CORONAVIRUS	VIRAL PROTEIN
2L5P	A	L-3N	98	203	69	69	1.086	1.299	1.404	0.005	0.097	-0.548	-0.290	0.304	RATTUS NORVEGICUS	TRANSPORT PROTEIN
5FI9	A	L-1N	225	248	143	143	0.589	0.703	0.795	0.007	0.098	-0.177	-0.524	0.305	MUS MUSCULUS	HYDROLASE/HYDROLASE INHIBITOR
2PSP	A	L-1C	8	35	7	7	0.550	0.818	0.898	0.019	0.096	-0.790	-0.462	0.306	SUS SCROFA	SIGNALING PROTEIN
1U3D	A	L+3N	80	190	67	67	0.980	1.612	1.730	0.026	0.097	-0.905	-0.277	0.306	ARABIDOPSIS THALIANA	SIGNALING PROTEIN
1M4L	A	L+1C	138	161	137	137	0.504	0.739	0.831	0.020	0.098	-0.678	-0.524	0.306	BOS TAURUS	HYDROLASE
5MS9	A	L-1N	195	221	84	84	0.564	0.744	0.759	0.008	0.097	-0.977	-0.476	0.306	HOMO SAPIENS	STRUCTURAL PROTEIN
5HJ3	C	L+1N	121	147	90	90	0.769	0.958	1.200	0.016	0.097	0.211	-0.476	0.308	EBOLA VIRUS	VIRAL PROTEIN
2ZX2	A	L-1C	106	135	105	105	0.642	1.114	1.182	0.030	0.096	-0.939	-0.435	0.310	ONCORHYNCHUS KETA	IMMUNE SYSTEM
4D0T	A	L+3N	345	423	270	270	1.190	1.266	1.533	0.006	0.100	0.799	-0.384	0.311	SYNTHETIC CONSTRUCT	TRANSFERASE
4HWM	A	L-2N	68	124	47	47	0.828	1.009	1.162	0.009	0.101	-0.151	-0.393	0.312	KLEBSIELLA PNEUMONIAE	UNKNOWN FUNCTION
5NTB	A	L+1C	23	53	22	22	0.555	0.910	0.994	0.027	0.096	-0.856	-0.423	0.313	STREPTOMYCES MOBARAENSIS	PROTEASE INHIBITOR
3RT4	A	L-2C	22	67	21	21	0.852	1.147	1.269	0.013	0.103	-0.647	-0.474	0.314	CAMELUS DROMEDARIUS	IMMUNE SYSTEM
5H4S	A	L-1C	198	229	197	197	0.634	1.182	1.234	0.036	0.095	-0.972	-0.412	0.316	TOXOPNEUSTES PILEOLUS	SUGAR BINDING PROTEIN
2JX9	A	L-1C	41	71	12	12	0.636	1.154	1.214	0.034	0.096	-0.960	-0.423	0.317	MUS MUSCULUS	CELL ADHESION, SIGNALING PROTEIN
1F56	A	L-1N	47	80	46	46	0.753	0.887	0.995	0.006	0.095	-0.177	-0.390	0.317	SPINACIA OLERACEA	PLANT PROTEIN
5G38	A	L-1C	19	44	0	0	0.691	0.983	1.176	0.022	0.097	-0.346	-0.491	0.318	THERMOSYNECHOCOCCUS ELONGATUS	PHOTOSYNTHESIS
1B7G	Q	L+1N	123	149	122	122	0.527	0.853	0.983	0.030	0.097	-0.668	-0.476	0.318	SULFOLOBUS SOLFATARICUS	OXIDOREDUCTASE
5Y32	B	L-2C	143	185	112	112	0.879	1.121	1.238	0.010	0.104	-0.561	-0.501	0.318	MUS MUSCULUS	IMMUNE SYSTEM
6H2T	A	L+3C	66	167	25	25	1.018	1.161	1.312	0.005	0.098	0.045	-0.302	0.319	MYCOBACTERIUM TUBERCULOSIS H37RV	SIGNALING PROTEIN
4JJO	A	L+1C	23	48	22	22	0.501	0.867	0.951	0.032	0.097	-0.862	-0.491	0.320	CLAVIBACTER MICHIGANENSIS	SUGAR BINDING PROTEIN
2JD4	B	L-1N	2845	2870	160	160	0.599	0.855	0.975	0.019	0.097	-0.581	-0.491	0.321	MUS MUSCULUS	METAL BINDING PROTEIN
1G0Y	R	L-2C	104	147	98	98	0.880	1.051	1.153	0.006	0.104	-0.421	-0.492	0.321	HOMO SAPIENS	IMMUNE SYSTEM
1SZN	A	L-1N	392	414	391	391	0.450	0.998	1.073	0.055	0.098	-0.944	-0.542	0.321	HYPOCREA JECORINA	HYDROLASE
4JP6	A	L+1C	29	61	28	28	0.590	0.930	1.021	0.024	0.095	-0.821	-0.401	0.325	CARICA PAPAYA	UNKNOWN FUNCTION
5H4S	A	L-1C	97	123	96	96	0.520	1.004	1.060	0.040	0.097	-0.961	-0.476	0.327	TOXOPNEUSTES PILEOLUS	SUGAR BINDING PROTEIN
4OIE	A	L-1C	280	329	104	104	0.692	0.995	1.010	0.013	0.094	-0.993	-0.267	0.328	WEST NILE VIRUS	VIRAL PROTEIN
3X2G	A	L+1C	26	54	25	25	0.556	0.791	0.876	0.017	0.096	-0.716	-0.448	0.330	PHANEROCHAETE CHRYSOSPORIUM	HYDROLASE
4JPH	A	L-1C	87	137	41	41	0.596	2.092	2.138	0.099	0.094	-0.997	-0.261	0.330	MUS MUSCULUS	CYTOKINE
1WS8	A	L-1N	58	92	57	57	0.712	0.920	1.042	0.012	0.095	-0.430	-0.380	0.331	CUCURBITA PEPO	ELECTRON TRANSPORT
1X9R	A	L-1N	57	91	57	57	0.712	0.925	1.031	0.011	0.095	-0.540	-0.380	0.331	ARMORACIA RUSTICANA	ELECTRON TRANSPORT
2PT5	A	L+1N	10	110	9	9	1.025	1.119	1.245	0.003	0.093	0.248	-0.096	0.333	AQUIFEX AEOLICUS	TRANSFERASE
2KD3	A	L-1C	69	123	21	21	0.790	1.814	1.920	0.057	0.093	-0.967	-0.240	0.333	MUS MUSCULUS	PROTEIN BINDING
3ES6	B	L-1N	61	95	60	60	0.692	1.330	1.375	0.038	0.095	-0.985	-0.380	0.333	HOMO SAPIENS	CELL ADHESION
1UAP	A	L-2C	34	101	10	10	0.942	1.021	1.231	0.007	0.099	0.692	-0.332	0.333	HOMO SAPIENS	PROTEIN BINDING
1SZN	A	L+1N	104	134	103	103	0.612	0.854	0.926	0.014	0.096	-0.791	-0.423	0.333	HYPOCREA JECORINA	HYDROLASE
4NT5	A	L-1C	2739	2788	18	18	0.708	1.863	1.913	0.069	0.094	-0.994	-0.267	0.334	HOMO SAPIENS	PROTEIN BINDING
2LT5	A	L+2C	3	78	2	2	0.940	1.205	1.233	0.007	0.098	-0.965	-0.296	0.334	RANA PIPIENS	HYDROLASE
2KKY	A	L-1N	141	177	51	51	0.643	0.850	0.905	0.010	0.095	-0.825	-0.361	0.335	ESCHERICHIA COLI	UNKNOWN FUNCTION
3ASI	A	L-1N	1015	1043	148	148	0.611	0.825	0.946	0.015	0.096	-0.456	-0.448	0.335	BOS TAURUS	CELL ADHESION
3JD6	O	L-1C	110	142	52	52	0.740	1.100	1.198	0.019	0.095	-0.819	-0.401	0.335	HOMO SAPIENS	CELL ADHESION
1UAP	A	L-2C	30	98	6	6	0.938	1.072	1.270	0.008	0.099	0.319	-0.327	0.336	HOMO SAPIENS	PROTEIN BINDING
3OP8	A	L-1N	288	326	45	45	0.681	1.090	1.225	0.027	0.094	-0.752	-0.343	0.336	HOMO SAPIENS	PROTEIN BINDING
1JER	A	L-1N	60	95	60	60	0.776	0.949	1.072	0.008	0.095	-0.290	-0.370	0.337	CUCUMIS SATIVUS	ELECTRON TRANSPORT
1D2B	A	L-2C	1	70	0	0	0.938	1.069	1.246	0.007	0.099	0.251	-0.322	0.337	HOMO SAPIENS	HYDROLASE INHIBITOR
5AEJ	A	L-1C	108	158	37	37	0.601	2.098	2.140	0.099	0.094	-0.997	-0.261	0.340	HOMO SAPIENS	SIGNALING PROTEIN
3UTK	A	L+1N	61	115	23	23	0.784	1.190	1.277	0.020	0.093	-0.880	-0.240	0.340	DICKEYA DADANTII	PROTEIN TRANSPORT
4A56	A	L+1N	53	107	23	23	0.775	1.178	1.255	0.019	0.093	-0.901	-0.240	0.340	KLEBSIELLA OXYTOCA	PROTEIN TRANSPORT

Continued on next page

Table S6 – *Continued from previous page*

code	chain	type	open	close	tail1	tail2	a	b	c	Asph	Pred_A	Prolat	Pred_P	motif_P	org	func
4K0U	A	L+1N	61	115	23	23	0.782	1.191	1.277	0.020	0.093	-0.882	-0.240	0.340	DICKEYA DADANTII	PROTEIN TRANSPORT
2HCZ	X	L+1C	42	70	38	38	0.486	0.907	0.964	0.037	0.096	-0.946	-0.448	0.340	ZEA MAYS	ALLERGEN
3LY9	A	L+1N	208	272	18	18	0.801	1.333	1.387	0.025	0.093	-0.969	-0.196	0.341	ESCHERICHIA COLI	SIGNALING PROTEIN
2ZX2	A	L-1C	6	35	5	5	0.624	1.120	1.195	0.033	0.096	-0.934	-0.435	0.342	ONCORHYNCHUS KETA	IMMUNE SYSTEM
6B7N	A	L-1C	361	386	309	309	0.585	0.847	0.893	0.015	0.097	-0.916	-0.491	0.343	DELTACORONAVIRUS	VIRAL PROTEIN
1O5E	L	L-1C	32	95	27	27	0.830	1.009	1.109	0.007	0.093	-0.470	-0.200	0.343	HOMO SAPIENS	SERINE PROTEASE, HYDROLASE
6EGT	A	L-3N	771	965	80	80	0.877	2.886	2.923	0.092	0.093	-0.999	-0.134	0.344	RIFT VALLEY FEVER VIRUS	VIRAL PROTEIN
1ZD0	A	LS2–N	48	131	44	44	0.883	1.109	1.206	0.008	0.097	-0.633	-0.266	0.344	PYROCOCCUS FURIOSUS	UNKNOWN FUNCTION
4CVW	C	L+1N	32	87	24	24	0.927	1.076	1.338	0.012	0.093	0.454	-0.235	0.345	HORDEUM VULGARE	HYDROLASE
4LQ6	A	L+1N	33	81	31	31	0.721	1.082	1.215	0.021	0.094	-0.705	-0.273	0.346	MYCOBACTERIUM TUBERCULOSIS	HYDROLASE
2KQA	A	L+1C	20	57	19	19	0.656	0.768	0.935	0.011	0.095	0.340	-0.352	0.347	CERATOCYSTIS PLATANI	TOXIN
3SUK	A	L+1C	39	76	22	22	0.638	0.782	0.933	0.012	0.095	0.038	-0.352	0.347	MONILIOPHTHORA PERNICIOSA	UNKNOWN FUNCTION
1R8O	A	L-1N	40	84	39	39	0.734	1.177	1.263	0.024	0.094	-0.898	-0.298	0.349	COPAIFERA LANGSDORFFII	HYDROLASE INHIBITOR
2LJM	A	L-1N	28	81	27	27	0.759	0.868	1.033	0.008	0.093	0.351	-0.245	0.349	CENTRUROIDES SUFFUSUS SUFFUSUS	TOXIN
1XWE	A	L-2C	1514	1588	6	6	0.951	1.191	1.313	0.008	0.098	-0.529	-0.301	0.350	HOMO SAPIENS	SIGNALING PROTEIN
1SGL	A	L-1C	57	90	53	53	0.786	0.930	1.128	0.011	0.095	0.263	-0.390	0.352	TRICHOSANTHES LEPINIANA	HYDROLASE
3X0H	A	L-1C	16	47	15	15	0.642	0.854	1.011	0.016	0.095	-0.253	-0.412	0.352	TALAROMYCES CELLULOLYTICUS CF-2612	HYDROLASE
5B5S	A	L-1C	16	47	15	15	0.642	0.854	1.011	0.016	0.095	-0.254	-0.412	0.352	TALAROMYCES CELLULOLYTICUS	HYDROLASE
3SUM	A	L+1C	43	80	21	21	0.595	0.877	0.959	0.018	0.095	-0.797	-0.352	0.354	MONILIOPHTHORA PERNICIOSA	UNKNOWN FUNCTION
1G66	A	L+1N	147	179	146	146	0.613	0.891	0.988	0.018	0.095	-0.723	-0.401	0.354	PENICILLIUM PURPUROGENUM	HYDROLASE
5XOZ	A	L-1N	53	98	41	41	0.770	1.193	1.302	0.022	0.094	-0.834	-0.292	0.355	CICER ARIETINUM	HYDROLASE INHIBITOR
5GNB	A	L+1N	485	516	174	174	0.450	1.119	1.172	0.065	0.095	-0.981	-0.412	0.355	HUMAN CORONAVIRUS	VIRAL PROTEIN
3NSJ	A	L-3N	241	407	220	220	1.619	1.846	2.356	0.013	0.094	0.609	-0.170	0.355	MUS MUSCULUS	IMMUNE SYSTEM
4H0W	A	LS3–+N	137	331	136	136	1.324	1.774	1.842	0.010	0.093	-0.934	-0.134	0.355	HOMO SAPIENS	METAL TRANSPORT
3FLP	A	L-1N	184	215	183	183	0.753	0.984	1.201	0.017	0.095	-0.053	-0.412	0.356	LIMULUS POLYPHEMUS	SUGAR BINDING PROTEIN
6GRF	B	L-1C	148	215	122	122	0.690	1.139	1.196	0.025	0.093	-0.953	-0.185	0.358	ARABIDOPSIS THALIANA	SIGNALING PROTEIN
5DSS	B	L-1N	45	90	44	44	0.786	1.150	1.259	0.018	0.094	-0.789	-0.292	0.358	MUCUNA PRURIENS	PLANT PROTEIN
5U81	A	L-3C	31	340	4	4	1.387	1.659	1.684	0.004	0.091	-0.973	-0.043	0.358	HETEROCEPHALUS GLABER	HYDROLASE
2OR7	A	L-1N	38	90	32	32	0.788	0.950	1.058	0.007	0.093	-0.329	-0.250	0.359	MUS MUSCULUS	IMMUNE SYSTEM
1BR9	A	L-2C	1	72	0	0	0.978	1.176	1.312	0.007	0.098	-0.317	-0.313	0.359	HOMO SAPIENS	PROTEINASE INHIBITOR
5H4S	A	L-1C	103	136	102	102	0.606	1.276	1.321	0.047	0.095	-0.986	-0.390	0.359	TOXOPNEUSTES PILEOLUS	SUGAR BINDING PROTEIN
5H4S	A	L-1C	6	36	5	5	0.631	1.147	1.212	0.034	0.096	-0.953	-0.423	0.359	TOXOPNEUSTES PILEOLUS	SUGAR BINDING PROTEIN
5MFA	A	L-3C	158	319	1	1	1.348	1.782	1.980	0.012	0.094	-0.596	-0.177	0.360	HOMO SAPIENS	OXIDOREDUCTASE
3QL6	A	L-3C	6	167	5	5	1.368	1.766	1.960	0.011	0.094	-0.556	-0.177	0.360	BOS TAURUS	OXIDOREDUCTASE
5K1E	A	L-3C	6	167	5	5	1.357	1.768	1.956	0.011	0.094	-0.594	-0.177	0.360	BOS TAURUS	OXIDOREDUCTASE
1HCN	B	L-1C	23	72	21	21	0.698	1.896	1.923	0.072	0.094	-0.998	-0.267	0.363	HOMO SAPIENS	HORMONE
5OP0	A	L-1N	89	128	82	82	0.643	1.014	1.065	0.021	0.094	-0.946	-0.335	0.363	MYCOBACTERIUM SMEGMATIS	TRANSFERASE
5BPU	A	L-1C	55	110	24	24	0.831	2.016	2.109	0.062	0.093	-0.981	-0.235	0.363	HOMO SAPIENS	SIGNALING PROTEIN
1Q25	A	L+1C	81	111	76	76	0.447	1.283	1.310	0.078	0.096	-0.996	-0.423	0.364	BOS TAURUS	PROTEIN TRANSPORT
2JON	A	L-1N	48	94	47	47	0.752	0.975	1.045	0.009	0.094	-0.771	-0.285	0.364	OLEA EUROPAEA	ALLERGEN
1FL7	B	L-1N	20	104	17	17	0.789	1.683	1.714	0.047	0.093	-0.996	-0.132	0.365	HOMO SAPIENS	HORMONE
3SH4	A	L-1N	159	193	158	158	0.562	1.149	1.193	0.044	0.095	-0.983	-0.380	0.365	HOMO SAPIENS	METAL BINDING PROTEIN
1AOC	A	L+2C	10	95	9	9	1.047	1.465	1.694	0.018	0.097	-0.483	-0.259	0.365	TACHYPLEUS TRIDENTATUS	COAGULATION FACTOR
4JGL	A	LL-1,-2	57	142	17	17	0.960	1.257	1.426	0.013	0.099	-0.456	-0.355	0.366	BACTEROIDES EGGERTHII	UNKNOWN FUNCTION
1ITU	A	L+1N	226	258	225	225	0.511	0.934	1.017	0.036	0.095	-0.896	-0.401	0.366	HOMO SAPIENS	HYDROLASE
1CFE	A	L-1C	44	112	43	43	0.804	1.117	1.189	0.013	0.093	-0.862	-0.181	0.367	SOLANUM LYCOPERSICUM	PATHOGENESIS-RELATED PROTEIN
1SVB	A	L-1C	74	105	73	73	0.528	1.154	1.187	0.050	0.095	-0.991	-0.412	0.367	TICK-BORNE ENCEPHALITIS VIRUS	VIRAL PROTEIN
4JHS	A	L-1N	307	345	103	103	0.705	1.055	1.207	0.023	0.094	-0.618	-0.343	0.367	HOMO SAPIENS	SIGNALING PROTEIN
2P3X	A	L-2C	25	88	24	24	1.004	1.270	1.474	0.012	0.100	-0.229	-0.352	0.367	VITIS VINIFERA	OXIDOREDUCTASE
1AOC	A	L-1C	60	161	59	59	0.848	1.457	1.505	0.028	0.093	-0.980	-0.094	0.367	TACHYPLEUS TRIDENTATUS	COAGULATION FACTOR
1FL7	B	L-1C	17	66	14	14	0.761	1.871	1.897	0.062	0.094	-0.998	-0.267	0.368	HOMO SAPIENS	HORMONE
6MEI	C	L-2C	429	503	19	19	1.024	1.436	1.658	0.018	0.098	-0.491	-0.301	0.369	HEPACIVIRUS C	IMMUNE SYSTEM

Continued on next page

Table S6 – *Continued from previous page*

code	chain	type	open	close	tail1	tail2	a	b	c	Asph	Pred_A	Prolat	Pred_P	motif_P	org	func
2JIG	A	L-1N	195	230	159	159	0.673	1.008	1.154	0.023	0.095	-0.619	-0.370	0.369	CHLAMYDOMONAS REINHARDTII	HYDROLASE
2JD4	B	L-1N	3024	3055	339	339	0.569	0.988	1.069	0.031	0.095	-0.900	-0.412	0.370	MUS MUSCULUS	METAL BINDING PROTEIN
5V50	A	L-1C	74	136	43	43	0.883	1.002	1.102	0.004	0.093	-0.151	-0.204	0.371	MONILIOPHTHORA PERNICIOSA	LIPID BINDING PROTEIN
5FFG	B	L+1C	235	276	122	122	0.791	1.273	1.431	0.027	0.094	-0.754	-0.320	0.371	HOMO SAPIENS	CELL ADHESION
5O6A	A	L-1C	74	105	73	73	0.545	1.148	1.174	0.046	0.095	-0.994	-0.412	0.371	TICK-BORNE ENCEPHALITIS VIRUS	VIRUS
4NDS	A	L-1C	2	68	1	1	0.743	1.099	1.101	0.015	0.093	-1.000	-0.188	0.372	LYOPHYLLUM DECASTES	SUGAR BINDING PROTEIN
1WC2	A	L+1C	30	69	29	29	0.644	0.960	1.090	0.022	0.094	-0.654	-0.335	0.373	MYTILUS EDULIS	HYDROLASE
3BQH	A	L-1N	207	349	10	10	1.100	1.199	1.304	0.002	0.093	0.041	-0.030	0.373	NEISSERIA MENINGITIDIS	OXIDOREDUCTASE
4Z11	A	L-2C	31	94	30	30	0.960	1.274	1.451	0.014	0.100	-0.465	-0.352	0.374	COREOPSIS GRANDIFLORA	OXIDOREDUCTASE
3A23	A	L+1N	148	188	103	103	0.652	1.038	1.090	0.022	0.094	-0.948	-0.327	0.374	STREPTOMYCES AVERMITILIS	HYDROLASE
5XBU	A	L+1C	33	73	33	33	0.628	0.944	1.067	0.022	0.094	-0.676	-0.327	0.375	AMPULLARIA CROSSEAN	HYDROLASE
4GQR	A	L+1N	70	115	69	69	0.656	1.323	1.438	0.046	0.094	-0.917	-0.292	0.377	HOMO SAPIENS	HYDROLASE
4A3X	A	LS2–N	180	262	140	140	1.062	1.266	1.447	0.008	0.097	-0.101	-0.270	0.377	CANDIDA GLABRATA	CELL ADHESION
4PLM	A	L-1C	121	154	81	81	0.711	1.214	1.264	0.028	0.095	-0.970	-0.390	0.377	GALLUS GALLUS	PROTEIN BINDING
1Q77	A	L+1N	14	114	16	16	0.877	1.322	1.469	0.021	0.093	-0.748	-0.096	0.377	AQUIFEX AEOLICUS	UNKNOWN FUNCTION
5A3L	A	LS2–N	152	239	119	119	0.958	1.554	1.652	0.024	0.097	-0.924	-0.252	0.378	KOMAGATAELLA PASTORIS	CELL ADHESION
3P06	A	L-2N	738	830	101	101	0.951	1.017	1.147	0.003	0.096	0.536	-0.237	0.378	TELLINA VIRUS 1	HYDROLASE
2VGA	A	L+2C	6	166	0	0	0.957	1.557	1.562	0.022	0.093	-1.000	-0.101	0.382	VACCINIA VIRUS	VIRAL PROTEIN
5I4H	B	L-1N	156	209	47	47	0.815	0.932	1.117	0.009	0.093	0.377	-0.245	0.382	HOMO SAPIENS	HYDROLASE
5JYS	A	L-1C	202	269	43	43	0.836	1.090	1.139	0.008	0.093	-0.901	-0.185	0.384	SACCHAROMYCES CEREVISIAE	TRANSPORT PROTEIN
5WA2	A	L-1N	71	192	14	14	0.948	1.201	1.287	0.008	0.093	-0.737	-0.059	0.385	TOXOPLASMA GONDII	MEMBRANE PROTEIN
5J81	A	L-1C	745	780	79	79	0.613	1.144	1.152	0.034	0.095	-0.999	-0.370	0.385	PUUMALA VIRUS (STRAIN P360)	VIRAL PROTEIN
2D7I	A	L+2N	356	432	288	288	1.201	1.314	1.565	0.006	0.098	0.603	-0.292	0.386	HOMO SAPIENS	TRANSFERASE
2FFU	A	L+2N	345	423	270	270	1.182	1.250	1.520	0.006	0.098	0.839	-0.284	0.387	HOMO SAPIENS	TRANSFERASE
1T61	A	L-1N	53	108	48	48	0.732	1.281	1.388	0.032	0.093	-0.898	-0.235	0.388	BOS TAURUS	STRUCTURAL PROTEIN
3MTW	A	L+1N	172	213	145	145	0.643	1.077	1.120	0.026	0.094	-0.969	-0.320	0.389	CAULOBACTER VIBRIOIDES	HYDROLASE
4A3X	A	LL-1,+1	78	119	38	38	1.002	1.106	1.320	0.007	0.104	0.562	-0.511	0.389	CANDIDA GLABRATA	CELL ADHESION
2XXL	A	L-1C	42	78	15	15	0.633	0.793	0.953	0.014	0.095	0.000	-0.361	0.389	DROSOPHILA MELANOGASTER	HYDROLASE
1IHN	A	L+1C	15	97	6	6	0.921	0.994	1.103	0.003	0.093	0.332	-0.138	0.390	METHANOTHERMOBACTER	UNKNOWN FUNCTION
4P27	A	L-1C	56	130	52	52	0.958	1.097	1.261	0.006	0.093	0.140	-0.161	0.390	SCHISTOSOMA MANSONI	ALLERGEN
3VX0	A	L-1N	440	475	439	439	0.752	0.886	1.096	0.012	0.095	0.374	-0.370	0.390	ASPERGILLUS ORYZAE	HYDROLASE
5IAZ	A	L-1C	732	785	6	6	0.907	1.381	1.570	0.024	0.093	-0.670	-0.245	0.391	ORYZA SATIVA SUBSP. INDICA	HYDROLASE
2ZK9	X	L-1N	77	126	76	76	0.592	1.178	1.188	0.040	0.094	-0.999	-0.267	0.391	CHRYSEOBACTERIUM PROTEOLYTICUM	HYDROLASE
5A3L	A	LS2–C	37	151	4	4	1.005	1.441	1.495	0.014	0.095	-0.955	-0.180	0.391	KOMAGATAELLA PASTORIS	CELL ADHESION
1HCN	B	L-1N	26	110	24	24	0.799	1.698	1.713	0.046	0.093	-0.999	-0.132	0.392	HOMO SAPIENS	HORMONE
6IWR	A	L+2N	426	507	314	314	1.246	1.351	1.654	0.007	0.097	0.730	-0.273	0.392	HOMO SAPIENS	TRANSFERASE
4HS9	A	L+1C	181	238	180	180	0.766	1.378	1.436	0.032	0.093	-0.972	-0.226	0.393	PROTEUS MIRABILIS	HYDROLASE
4OMB	A	L+1N	153	197	128	128	0.677	1.193	1.305	0.033	0.094	-0.875	-0.298	0.394	PSEUDOMONAS AERUGINOSA	TRANSPORT PROTEIN
3QVP	A	L+1N	164	206	161	161	0.811	1.173	1.398	0.023	0.094	-0.394	-0.312	0.395	ASPERGILLUS NIGER	OXIDOREDUCTASE
1C1Z	A	L-1N	288	326	287	287	0.708	1.045	1.200	0.022	0.094	-0.590	-0.343	0.396	HOMO SAPIENS	SIGNALING PROTEIN
5EX2	A	L-2N	150	252	150	150	1.187	1.368	1.534	0.005	0.096	-0.078	-0.208	0.396	HIRSCHIA BALTICA	ISOMERASE
4YGF	A	LS2++C	45	195	23	23	1.135	1.322	1.368	0.003	0.093	-0.846	-0.115	0.396	HELICOBACTER PYLORI	LYASE
2KQA	A	L+1N	60	115	59	59	0.773	0.922	0.982	0.005	0.093	-0.661	-0.235	0.396	CERATOCYSTIS PLATANI	TOXIN
5Y10	C	L-1C	180	327	157	157	0.857	2.067	2.111	0.060	0.092	-0.996	-0.024	0.397	THROMBOCYTOPENIA VIRUS	VIRAL PROTEIN
5GYQ	A	L-1C	50	103	47	47	0.710	1.148	1.170	0.022	0.093	-0.992	-0.245	0.397	BAT CORONAVIRUS HKU9	VIRAL PROTEIN
4IJD	A	L+1N	351	394	156	156	0.734	1.308	1.455	0.036	0.094	-0.837	-0.305	0.397	HOMO SAPIENS	TRANSFERASE
2YAU	A	L+2N	89	213	88	88	1.120	1.712	1.880	0.022	0.094	-0.805	-0.159	0.397	LEISHMANIA INFANTUM	OXIDOREDUCTASE
2K8P	A	L-1C	70	124	69	69	0.590	2.225	2.244	0.106	0.093	-1.000	-0.240	0.398	HOMO SAPIENS	SIGNALING PROTEIN
1I1J	A	L-1N	35	106	34	34	0.851	1.046	1.101	0.006	0.093	-0.806	-0.171	0.398	HOMO SAPIENS	HORMONE
3UYX	A	L-1C	96	142	42	42	0.950	1.281	1.482	0.016	0.094	-0.410	-0.285	0.399	INFLUENZA A VIRUS	VIRAL PROTEIN
3MN8	C	L+1N	268	308	238	238	0.645	1.060	1.091	0.024	0.094	-0.982	-0.327	0.399	DROSOPHILA MELANOGASTER	HYDROLASE
1ZH1	A	L-1N	142	190	106	106	0.793	1.096	1.205	0.014	0.094	-0.712	-0.273	0.399	HEPATITIS C VIRUS	METAL BINDING PROTEIN

Continued on next page

Table S6 – *Continued from previous page*

code	chain	type	open	close	tail1	tail2	a	b	c	Asph	Pred_A	Prolat	Pred_P	motif_P	org	func
4FNK	A	L-1C	97	139	88	88	0.923	1.237	1.391	0.014	0.094	-0.550	-0.312	0.399	INFLUENZA A VIRUS	VIRAL PROTEIN
1FLC	A	L-1C	196	238	195	195	0.647	1.624	1.712	0.066	0.094	-0.975	-0.312	0.400	INFLUENZA C VIRUS	HYDROLASE
4A3X	A	LS2–C	50	179	10	10	1.001	1.497	1.546	0.017	0.094	-0.970	-0.149	0.400	CANDIDA GLABRATA	CELL ADHESION
5Y0W	A	L-1C	151	303	150	150	0.907	2.136	2.208	0.058	0.092	-0.989	-0.019	0.400	RIFT VALLEY FEVER VIRUS	VIRAL PROTEIN
1AX8	A	L+1N	96	146	93	93	0.836	1.214	1.426	0.022	0.094	-0.466	-0.261	0.400	HOMO SAPIENS	CYTOKINE
4JP6	A	L+1N	64	120	63	63	0.737	0.895	0.985	0.007	0.093	-0.450	-0.230	0.401	CARICA PAPAYA	UNKNOWN FUNCTION
3S4O	A	L+1N	51	112	50	50	0.856	1.133	1.319	0.015	0.093	-0.329	-0.208	0.402	LEISHMANIA MAJOR	UNKNOWN FUNCTION
2GGO	A	L+1N	44	109	43	43	0.880	1.079	1.152	0.006	0.093	-0.706	-0.192	0.402	SULFOLOBUS TOKODAII	TRANSFERASE
2MCF	A	L-1N	24	120	23	23	0.878	1.139	1.172	0.008	0.093	-0.955	-0.104	0.402	THERMOCOCCUS GAMMATOLERANS	UNKNOWN FUNCTION
2GRK	A	L+2C	13	192	5	5	1.152	1.447	1.579	0.008	0.092	-0.608	-0.077	0.402	ECTROMELIA VIRUS	VIRAL PROTEIN
3LPA	A	L+1N	89	141	87	87	0.642	1.463	1.540	0.056	0.093	-0.973	-0.250	0.403	DICHELOBACTER NODOSUS	HYDROLASE
1FJR	A	L-1C	70	164	69	69	0.991	1.148	1.234	0.004	0.093	-0.478	-0.108	0.403	DROSOPHILA MELANOGASTER	SIGNALING PROTEIN
1JLI	A	L+1C	16	84	2	2	0.921	1.031	1.158	0.004	0.093	0.127	-0.181	0.404	HOMO SAPIENS	CYTOKINE
2L3O	A	L+1C	43	106	10	10	0.882	0.997	1.077	0.003	0.093	-0.311	-0.200	0.405	MUS MUSCULUS	CYTOKINE
3SUK	A	L+1N	79	138	62	62	0.759	0.938	1.038	0.008	0.093	-0.466	-0.217	0.407	MONILIOPHTHORA PERNICIOSA	UNKNOWN FUNCTION
4D6W	B	L-1N	178	223	177	177	0.703	1.143	1.230	0.025	0.094	-0.894	-0.292	0.407	CHANDIPURA VIRUS	VIRAL PROTEIN
2QRL	A	L+1N	205	249	202	202	0.658	0.979	1.058	0.019	0.094	-0.845	-0.298	0.408	SACCHAROMYCES CEREVISIAE	OXIDOREDUCTASE
4HYQ	A	L+1N	152	199	151	151	0.595	1.208	1.313	0.046	0.094	-0.919	-0.279	0.408	STREPTOMYCES ALBIDOFLAVUS	HYDROLASE
5EX1	A	L-2N	147	270	138	138	1.272	1.631	1.759	0.009	0.094	-0.720	-0.161	0.408	HIRSCHIA BALTICA	ISOMERASE
3HEI	B	L-1N	80	140	62	62	0.919	1.196	1.335	0.011	0.093	-0.540	-0.212	0.408	HOMO SAPIENS	TRANSFERASE
2ZG2	A	L+1N	41	175	17	17	0.962	1.870	1.959	0.040	0.093	-0.971	-0.040	0.410	HOMO SAPIENS	IMMUNE SYSTEM
5LFR	A	L+1N	37	165	18	18	0.942	1.759	1.840	0.036	0.093	-0.970	-0.048	0.410	MUS MUSCULUS	CELL ADHESION
3SUM	A	L+1N	83	145	61	61	0.767	0.948	1.040	0.008	0.093	-0.532	-0.204	0.412	MONILIOPHTHORA PERNICIOSA	UNKNOWN FUNCTION
2CMZ	B	L-1N	177	224	176	176	0.750	1.073	1.132	0.014	0.094	-0.908	-0.279	0.413	INDIANA VIRUS	MEMBRANE PROTEIN
2KXI	A	L-1N	67	128	66	66	0.931	1.011	1.216	0.006	0.093	0.683	-0.208	0.413	NEISSERIA MENINGITIDIS	TRANSFERASE
5UBJ	A	L+2C	24	295	1	1	1.363	1.383	1.496	0.001	0.090	0.908	-0.000	0.413	EMERICELLA NIDULANS	HYDROLASE
4L3N	A	L-1C	425	478	45	45	0.729	1.128	1.147	0.018	0.093	-0.992	-0.245	0.413	HUMAN BETACORONAVIRUS	VIRAL PROTEIN
5WM0	A	L-1C	81	126	35	35	0.847	1.046	1.240	0.012	0.094	-0.024	-0.292	0.414	RATTUS NORVEGICUS	OXIDOREDUCTASE
3WMZ	A	L+2C	176	444	2	2	1.356	1.381	1.498	0.001	0.090	0.883	-0.002	0.414	STREPTOMYCES COELICOLOR	HYDROLASE
1DP4	A	L+1N	164	213	163	163	0.683	1.018	1.160	0.022	0.094	-0.634	-0.267	0.416	RATTUS NORVEGICUS	HORMONE
1IKO	P	L-1N	92	156	62	62	0.845	1.182	1.290	0.015	0.093	-0.759	-0.196	0.416	MUS MUSCULUS	SIGNALING PROTEIN
4F23	A	L-1C	95	138	94	94	0.938	1.273	1.450	0.015	0.094	-0.506	-0.305	0.417	INFLUENZA A VIRUS	VIRAL PROTEIN
5J83	A	L+1N	59	127	54	54	0.768	1.180	1.348	0.025	0.093	-0.653	-0.181	0.417	RHIZOBIUM LEGUMINOSARUM	LYASE
3A56	A	L-1N	191	240	173	173	0.596	1.175	1.183	0.039	0.094	-0.999	-0.267	0.417	CHRYSEOBACTERIUM PROTEOLYTICUM	HYDROLASE
4TPV	A	L-1C	61	129	58	58	0.813	1.080	1.110	0.009	0.093	-0.962	-0.181	0.418	ANCYLOSTOMA CANINUM	BLOOD CLOTTING
3QSD	A	L-1N	133	199	62	62	0.861	1.193	1.324	0.015	0.093	-0.672	-0.188	0.419	SCHISTOSOMA MANSONI	HYDROLASE
1DYS	B	L+1N	93	152	90	90	0.759	1.268	1.408	0.030	0.093	-0.815	-0.217	0.419	HUMICOLA INSOLENS	CELLULASE
1LE6	A	L-1N	48	122	47	47	0.947	1.259	1.453	0.015	0.093	-0.387	-0.161	0.420	HOMO SAPIENS	HYDROLASE
3ON9	A	L-1N	180	317	19	19	1.033	1.302	1.316	0.006	0.093	-0.991	-0.036	0.421	ECTROMELIA VIRUS	VIRAL PROTEIN
2RFT	A	L-1C	94	143	93	93	0.965	1.254	1.400	0.011	0.094	-0.533	-0.267	0.421	INFLUENZA B VIRUS	VIRAL PROTEIN
4XUU	A	L+1C	35	100	34	34	0.929	1.143	1.361	0.012	0.093	0.013	-0.192	0.421	HOMO SAPIENS	PROTEIN BINDING
3TNX	A	L-1N	260	307	250	250	0.714	0.919	1.049	0.012	0.094	-0.375	-0.279	0.422	CARICA PAPAYA	HYDROLASE
3I26	A	L-1C	108	156	92	92	1.057	1.224	1.567	0.014	0.094	0.552	-0.273	0.422	BREDA VIRUS SEROTYPE 1	HYDROLASE
2CMZ	B	L+1C	68	114	67	67	0.644	1.552	1.624	0.061	0.094	-0.981	-0.285	0.422	VESICULAR STOMATITIS INDIANA VIRUS	MEMBRANE PROTEIN
4D6W	B	L+1C	68	114	67	67	0.649	1.533	1.600	0.059	0.094	-0.982	-0.285	0.423	CHANDIPURA VIRUS	VIRAL PROTEIN
153L	A	L+1C	4	60	3	3	0.808	1.181	1.357	0.021	0.093	-0.574	-0.230	0.424	ANSER ANSER ANSER	HYDROLASE(O-GLYCOSYL)
3HWN	C	L-1N	156	209	155	155	0.827	0.928	1.101	0.007	0.093	0.434	-0.245	0.424	HOMO SAPIENS	HYDROLASE
1FLC	A	L-1C	126	174	125	125	0.957	1.304	1.547	0.018	0.094	-0.302	-0.273	0.425	INFLUENZA C VIRUS	HYDROLASE
2HCZ	X	L+1N	73	140	69	69	0.806	0.965	1.046	0.006	0.093	-0.532	-0.185	0.425	ZEA MAYS	ALLERGEN
5VKJ	A	L+1N	39	167	22	22	1.019	1.794	1.818	0.029	0.093	-0.997	-0.048	0.426	HOMO SAPIENS	IMMUNE SYSTEM
5UBL	A	L-1C	593	674	592	592	1.049	1.146	1.468	0.011	0.093	0.787	-0.140	0.426	PSEUDOMONAS AERUGINOSA	HYDROLASE
1NYO	A	L-1C	8	142	7	7	1.053	1.170	1.239	0.002	0.093	-0.429	-0.040	0.427	MYCOBACTERIUM TUBERCULOSIS	IMMUNE SYSTEM

Continued on next page

Table S6 – *Continued from previous page*

code	chain	type	open	close	tail1	tail2	a	b	c	Asph	Pred_A	Prolat	Pred_P	motif_P	org	func
4HJ1	A	L-1C	777	825	89	89	0.678	1.138	1.185	0.026	0.094	-0.969	-0.273	0.428	RIFT VALLEY FEVER VIRUS	VIRAL PROTEIN
1ETE	A	L-1N	44	127	43	43	0.900	1.126	1.200	0.007	0.093	-0.754	-0.135	0.428	HOMO SAPIENS	CYTOKINE
1SGL	A	L-1C	26	84	22	22	0.889	1.298	1.425	0.018	0.093	-0.773	-0.221	0.428	TRICHOSANTHES LEPINIANA	HYDROLASE
1EXZ	C	L-1N	443	538	34	34	0.851	1.349	1.371	0.020	0.093	-0.994	-0.106	0.428	HOMO SAPIENS	HORMONE
6EGT	A	L-1C	777	825	86	86	0.678	1.155	1.197	0.027	0.094	-0.976	-0.273	0.429	RIFT VALLEY FEVER VIRUS	VIRAL PROTEIN
1KKH	A	L-2N	112	286	111	111	1.167	1.470	1.585	0.008	0.092	-0.692	-0.083	0.429	METHANOCALDOCOCCUS JANNASCHII	TRANSFERASE
1GAK	A	L-1N	60	134	59	59	0.827	1.467	1.595	0.034	0.093	-0.892	-0.161	0.430	HALIOTIS FULGENS	CELL ADHESION
3QW9	A	L-1N	84	153	73	73	0.934	1.451	1.531	0.021	0.093	-0.933	-0.178	0.430	RATTUS NORVEGICUS	CYTOKINE RECEPTOR
4I05	A	L-1N	133	199	86	86	0.863	1.195	1.322	0.015	0.093	-0.685	-0.188	0.431	SCHISTOSOMA MANSONI	HYDROLASE
4YFA	A	L-1C	49	138	37	37	1.096	1.138	1.457	0.009	0.093	0.949	-0.120	0.431	ACIDOVORAX SP. MR-S7	HYDROLASE
6EIB	A	L-1C	6	72	5	5	0.703	1.426	1.458	0.043	0.093	-0.994	-0.188	0.431	VIBRIO CHOLERAE	TRANSFERASE
3L49	A	L+1N	166	224	138	138	0.873	0.988	1.243	0.011	0.093	0.602	-0.221	0.431	RHODOBACTER SPHAEROIDES	TRANSPORT PROTEIN
4N3T	A	L-1N	87	162	61	61	0.921	1.068	1.118	0.003	0.093	-0.735	-0.158	0.432	CANDIDA ALBICANS	OXIDOREDUCTASE
2WBF	X	L-1N	755	809	192	192	0.817	1.023	1.171	0.010	0.093	-0.278	-0.240	0.432	PLASMODIUM FALCIPARUM	HYDROLASE
5X4R	A	LS2–C	30	195	12	12	1.253	1.588	1.790	0.010	0.093	-0.410	-0.094	0.433	CORONAVIRUS	VIRAL PROTEIN
2D1G	A	LL+1,+1	216	269	211	211	0.843	1.259	1.395	0.020	0.101	-0.755	-0.412	0.434	FRANCISELLA TULARENSIS	HYDROLASE
1S4Q	A	L+1N	40	193	20	20	1.187	1.476	1.592	0.007	0.092	-0.664	-0.018	0.435	MYCOBACTERIUM TUBERCULOSIS	TRANSFERASE
1T61	A	L-1N	164	222	159	159	0.734	1.427	1.447	0.038	0.093	-0.997	-0.221	0.436	BOS TAURUS	STRUCTURAL PROTEIN
4PMK	B	L+1C	56	148	8	8	1.025	1.104	1.223	0.003	0.093	0.337	-0.113	0.436	ACTINIDIA CHINENSIS	PLANT PROTEIN
3NT8	A	L-1C	291	377	267	267	0.931	1.208	1.283	0.009	0.093	-0.816	-0.127	0.436	NECATOR AMERICANUS	IMMUNE SYSTEM
4YKK	B	L-1N	200	255	199	199	0.894	1.471	1.615	0.028	0.093	-0.843	-0.235	0.436	MNEMIOPSIS LEIDYI	MEMBRANE PROTEIN
3ZK4	A	L+2N	203	367	181	181	1.232	1.528	1.644	0.007	0.093	-0.678	-0.095	0.437	LUPINUS LUTEUS	OXIDOREDUCTASE
1SFS	A	L+1N	21	200	17	17	1.116	1.307	1.460	0.006	0.092	-0.190	0.008	0.437	GEOBACILLUS STEAROTHERMOPHILUS	UNKNOWN FUNCTION
4BOE	A	L-1N	28	150	27	27	1.136	1.192	1.335	0.002	0.093	0.684	-0.057	0.437	RHIPICEPHALUS APPENDICULATUS	CHOLESTEROL BINDING PROTEIN
5GNB	A	L-1C	370	423	59	59	0.692	1.208	1.221	0.028	0.093	-0.998	-0.245	0.437	HUMAN CORONAVIRUS	VIRAL PROTEIN
5FV5	A	L+1C	37	167	21	21	0.912	1.370	1.492	0.020	0.093	-0.824	-0.046	0.437	KOMAGATAELLA PASTORIS	UNKNOWN FUNCTION
2X46	A	L-1N	50	155	34	34	1.055	1.118	1.254	0.003	0.093	0.583	-0.086	0.438	ARGAS REFLEXUS	ALLERGEN
2DVZ	A	LL+1,-1	93	152	78	78	0.876	1.646	1.783	0.039	0.100	-0.912	-0.374	0.438	BORDETELLA PERTUSSIS	TRANSPORT PROTEIN
3Q2U	A	L-1C	75	156	53	53	1.029	1.092	1.307	0.005	0.093	0.790	-0.140	0.438	HOMO SAPIENS	MEMBRANE PROTEIN
2ERF	A	L-1N	153	214	146	146	1.066	1.269	1.393	0.006	0.093	-0.405	-0.208	0.439	HOMO SAPIENS	SUGAR BINDING PROTEIN
4Z11	A	L+2C	206	445	205	205	1.410	1.786	1.844	0.007	0.091	-0.934	-0.022	0.439	COREOPSIS GRANDIFLORA	OXIDOREDUCTASE
6FPG	E	L+1C	64	157	23	23	1.038	1.137	1.254	0.003	0.093	0.149	-0.110	0.439	USTILAGO MAYDIS	CELL INVASION
2X97	A	L-2N	467	612	450	450	1.232	1.709	1.978	0.018	0.093	-0.459	-0.122	0.441	DROSOPHILA MELANOGASTER	HYDROLASE
1ESC	A	L+1N	197	255	193	193	0.672	1.354	1.446	0.044	0.093	-0.947	-0.221	0.441	STREPTOMYCES SCABIEI	HYDROLASE (SERINE ESTERASE)
3X2G	A	L+1N	57	142	56	56	0.941	1.038	1.182	0.004	0.093	0.330	-0.130	0.442	PHANEROCHAETE CHRYSOSPORIUM	HYDROLASE
3ZUI	A	L-1N	56	168	33	33	1.079	1.199	1.254	0.002	0.093	-0.589	-0.073	0.442	ORNITHODOROS MOUBATA	IMMUNE SYSTEM
4GQZ	C	L-1N	96	172	77	77	0.878	1.002	1.192	0.008	0.093	0.353	-0.155	0.442	SALMONELLA ENTERICA	METAL BINDING PROTEIN
6ANY	A	L-1C	82	170	65	65	0.903	1.282	1.371	0.015	0.093	-0.856	-0.122	0.442	BRUGIA MALAYI	LIPID TRANSPORT
1AVG	I	L-1N	39	142	38	38	1.025	1.073	1.181	0.002	0.093	0.612	-0.090	0.442	BOS TAURUS	BLOOD COAGULATION
2Z4I	B	L+1N	145	211	124	124	0.819	1.073	1.131	0.009	0.093	-0.864	-0.188	0.442	ESCHERICHIA COLI	SIGNALING PROTEIN ACTIVATOR
3WMT	A	L+1C	76	129	40	40	0.868	1.178	1.281	0.013	0.093	-0.749	-0.245	0.445	ASPERGILLUS ORYZAE	HYDROLASE
5H4U	A	L+1C	19	87	18	18	0.978	1.051	1.263	0.006	0.093	0.731	-0.181	0.445	CRYPTOPYGUS ANTARCTICUS	HYDROLASE
1F8R	A	L+2C	10	173	6	6	1.299	2.018	2.241	0.024	0.093	-0.775	-0.097	0.446	CALLOSELASMA RHODOSTOMA	OXIDOREDUCTASE
4UYS	A	L+1C	44	179	14	14	0.940	1.366	1.502	0.018	0.093	-0.764	-0.039	0.446	SACCHAROMYCES CEREVISIAE	CELL ADHESION
3T94	A	L+1N	138	205	137	137	0.924	1.220	1.397	0.014	0.093	-0.423	-0.185	0.447	SULFOLOBUS SOLFATARICUS	TRANSFERASE
1GXY	A	L+1N	21	223	17	17	1.146	1.442	1.489	0.006	0.092	-0.929	0.026	0.447	RATTUS NORVEGICUS	TRANSFERASE
1FOB	A	L+1N	253	311	252	252	0.850	1.101	1.223	0.011	0.093	-0.556	-0.221	0.448	ASPERGILLUS ACULEATUS	HYDROLASE
1XTA	A	L-1C	56	134	54	54	0.987	1.111	1.276	0.006	0.093	0.245	-0.149	0.449	NAJA ATRA	TOXIN
2ENG	A	L+1C	16	86	15	15	0.984	1.139	1.334	0.008	0.093	0.194	-0.174	0.449	HUMICOLA INSOLENS	HYDROLASE (ENDOGLUCANASE)
1WC2	A	L+1N	72	157	71	71	0.925	1.068	1.117	0.003	0.093	-0.737	-0.130	0.451	MYTILUS EDULIS	HYDROLASE
4WRN	A	L-1N	627	682	609	609	0.678	1.251	1.314	0.035	0.093	-0.964	-0.235	0.451	ESCHERICHIA COLI	STRUCTURAL PROTEIN
4OPB	A	L-1C	84	185	83	83	0.992	1.453	1.552	0.017	0.093	-0.879	-0.094	0.452	ASPERGILLUS ORYZAE	METAL BINDING PROTEIN

Continued on next page

Table S6 – *Continued from previous page*

code	chain	type	open	close	tail1	tail2	a	b	c	Asph	Pred_A	Prolat	Pred_P	motif_P	org	func
5H9K	A	L-1N	45	155	42	42	0.986	1.196	1.279	0.006	0.093	-0.671	-0.077	0.454	RHODNIUS PROLIXUS	PROTEIN BINDING
5XBU	A	L+1N	76	162	76	76	0.925	1.077	1.126	0.003	0.093	-0.766	-0.127	0.454	AMPULLARIA CROSSEAN	HYDROLASE
3PIW	A	L+1C	6	101	0	0	0.952	1.277	1.426	0.013	0.093	-0.592	-0.106	0.454	DANIO RERIO	CYTOKINE
3NGW	A	L+1N	24	192	23	23	1.175	1.232	1.411	0.003	0.092	0.767	-0.002	0.455	ARCHAEOGLOBUS FULGIDUS	BIOSYNTHETIC PROTEIN
3VUP	A	L+1N	177	244	176	176	0.758	1.181	1.233	0.020	0.093	-0.955	-0.185	0.455	APLYSIA KURODAI	HYDROLASE
3PIV	A	L+1C	4	99	0	0	0.921	1.245	1.346	0.012	0.093	-0.770	-0.106	0.456	DANIO RERIO	CYTOKINE
2JD4	B	LS2–C	2686	2958	1	1	1.183	2.157	2.243	0.033	0.090	-0.976	0.000	0.456	MUS MUSCULUS	METAL BINDING PROTEIN
5GZA	A	L-1C	72	139	19	19	0.889	0.995	1.103	0.004	0.093	0.022	-0.185	0.459	DANIO RERIO	TRANSFERASE
2WB9	A	L+1N	26	196	24	24	1.226	1.420	1.696	0.009	0.092	0.300	-0.001	0.459	FASCIOLA HEPATICA	TRANSFERASE
3HHC	B	L+1C	4	103	0	0	1.000	1.246	1.400	0.009	0.093	-0.381	-0.098	0.460	HOMO SAPIENS	CYTOKINE
1KI0	A	L-1C	169	297	88	88	1.016	1.611	1.682	0.022	0.093	-0.958	-0.048	0.460	HOMO SAPIENS	HYDROLASE
2HZQ	A	L-1N	41	165	38	38	1.171	1.226	1.344	0.002	0.093	0.587	-0.054	0.460	HOMO SAPIENS	TRANSPORT PROTEIN
4HKJ	D	L-1N	34	183	29	29	1.148	1.252	1.440	0.004	0.092	0.471	-0.022	0.461	MUS MUSCULUS	IMMUNE SYSTEM
5ZE3	A	L-1C	464	530	142	142	0.869	0.965	1.092	0.004	0.093	0.240	-0.188	0.461	HOMO SAPIENS	OXIDOREDUCTASE
4H0W	A	LS2–N	474	665	473	473	1.339	1.552	1.664	0.004	0.092	-0.504	-0.064	0.461	HOMO SAPIENS	METAL TRANSPORT
1MJN	A	L+1N	161	299	33	33	1.043	1.291	1.303	0.005	0.093	-0.993	-0.035	0.462	HOMO SAPIENS	IMMUNE SYSTEM
1LKI	A	L+1C	18	131	9	9	0.911	1.378	1.472	0.019	0.093	-0.892	-0.072	0.464	MUS MUSCULUS	CYTOKINE
2ZK9	X	L-1N	76	172	75	75	1.149	1.224	1.341	0.002	0.093	0.363	-0.104	0.465	CHRYSEOBACTERIUM PROTEOLYTICUM	HYDROLASE
4R2B	A	L-1N	290	357	263	263	0.779	1.409	1.520	0.035	0.093	-0.914	-0.185	0.465	OCHROBACTRUM ANTHROPI	TRANSPORT PROTEIN
2VGA	A	L-1N	33	199	27	27	1.072	1.487	1.520	0.011	0.092	-0.980	-0.004	0.466	VACCINIA VIRUS	VIRAL PROTEIN
1PZS	A	L-1N	54	165	53	53	1.136	1.294	1.324	0.002	0.093	-0.899	-0.075	0.466	MYCOBACTERIUM TUBERCULOSIS	OXIDOREDUCTASE
6E8N	A	L-1N	113	210	75	75	1.119	1.349	1.595	0.010	0.093	0.062	-0.102	0.466	HOMO SAPIENS	LIPID BINDING PROTEIN
4XVH	A	L-1C	154	326	130	130	1.188	1.380	1.415	0.003	0.092	-0.905	0.001	0.466	CHAETOMIUM OLIVICOLOR	HYDROLASE
5EJB	B	L+1N	71	192	45	45	1.001	1.792	1.946	0.034	0.093	-0.898	-0.059	0.467	HENDRA VIRUS	VIRAL PROTEIN
1LKI	A	L+1C	12	134	3	3	0.931	1.439	1.522	0.020	0.093	-0.926	-0.057	0.468	MUS MUSCULUS	CYTOKINE
1QFT	A	L-1N	48	169	47	47	1.113	1.169	1.295	0.002	0.093	0.620	-0.059	0.469	RHIPICEPHALUS APPENDICULATUS	LIGAND BINDING PROTEIN
1GKA	B	L-1N	46	170	45	45	1.133	1.207	1.304	0.002	0.093	0.243	-0.054	0.469	HOMARUS GAMMARUS	LIPOCALIN
4N7C	A	L-1N	44	175	41	41	1.120	1.187	1.335	0.003	0.093	0.598	-0.044	0.469	BLATTELLA GERMANICA	PROTEIN BINDING
2CVB	A	L+1N	45	173	43	43	0.985	1.245	1.273	0.006	0.093	-0.965	-0.048	0.470	THERMUS THERMOPHILUS	UNKNOWN FUNCTION
2FAW	A	L-1N	173	251	168	168	0.951	1.110	1.261	0.007	0.093	-0.043	-0.149	0.470	CARICA PAPAYA	TRANSFERASE
3PXL	A	L-2N	85	488	84	84	1.441	1.850	1.911	0.007	0.089	-0.937	0.061	0.470	TRAMETES HIRSUTA	OXIDOREDUCTASE
4GE1	A	L-1N	42	176	40	40	1.134	1.224	1.382	0.003	0.093	0.454	-0.040	0.470	RHODNIUS PROLIXUS	AMINE-BINDING PROTEIN
4RL4	B	L+1N	66	172	67	67	0.900	1.404	1.495	0.021	0.093	-0.910	-0.084	0.470	HELICOBACTER PYLORI	HYDROLASE
5L7R	A	LSL2–,+2	117	430	116	116	1.469	2.100	2.207	0.014	0.092	-0.920	-0.113	0.471	BACTEROIDES VULGATUS	HYDROLASE
5XBU	A	L+1N	69	175	69	69	1.024	1.131	1.289	0.004	0.093	0.330	-0.084	0.471	AMPULLARIA CROSSEAN	HYDROLASE
5YSC	A	L+1N	187	267	165	165	0.919	1.118	1.224	0.007	0.093	-0.505	-0.143	0.472	VIBRIO CHOLERAE O395	TRANSPORT PROTEIN
6FON	A	L-1N	141	227	133	133	0.969	1.038	1.117	0.002	0.093	0.106	-0.127	0.473	HOMO SAPIENS	METAL BINDING PROTEIN
2ENG	A	L+1N	89	189	88	88	0.899	1.224	1.302	0.012	0.093	-0.851	-0.096	0.474	HUMICOLA INSOLENS	HYDROLASE (ENDOGLUCANASE)
1WC2	A	L+1N	65	178	64	64	1.027	1.141	1.309	0.005	0.093	0.325	-0.072	0.475	MYTILUS EDULIS	HYDROLASE
4K6L	G	L-1N	56	207	37	37	1.117	1.392	1.613	0.011	0.092	-0.186	-0.020	0.477	SALMONELLA ENTERICA	TOXIN
5H4U	A	L+1N	90	193	89	89	0.923	1.210	1.298	0.010	0.093	-0.779	-0.090	0.477	CRYPTOPYGUS ANTARCTICUS	HYDROLASE
1FZG	E	L+1C	201	286	37	37	0.946	1.116	1.232	0.006	0.093	-0.314	-0.130	0.479	HOMO SAPIENS	BLOOD COAGULATION
3NT8	A	L-1C	89	167	65	65	0.866	1.245	1.337	0.016	0.093	-0.849	-0.149	0.481	NECATOR AMERICANUS	IMMUNE SYSTEM
6E8N	A	L-1N	88	222	50	50	1.107	1.596	1.698	0.015	0.093	-0.886	-0.040	0.481	HOMO SAPIENS	LIPID BINDING PROTEIN
4R72	A	L-1N	127	223	126	126	0.858	1.095	1.133	0.007	0.093	-0.930	-0.104	0.482	ACTINOBACILLUS PLEUROPNEUMONIAE	TRANSPORT PROTEIN
3EDY	A	L-1C	365	526	345	345	1.237	1.385	1.580	0.005	0.092	0.234	-0.009	0.483	HOMO SAPIENS	HYDROLASE
4GWN	A	L+1N	103	255	41	41	1.054	1.341	1.451	0.009	0.092	-0.685	-0.019	0.483	HOMO SAPIENS	HYDROLASE
2ENG	A	L+1N	87	199	86	86	0.998	1.225	1.329	0.007	0.093	-0.587	-0.073	0.484	HUMICOLA INSOLENS	HYDROLASE (ENDOGLUCANASE)
2VEC	A	L-1C	10	204	3	3	1.234	1.345	1.569	0.005	0.092	0.542	0.020	0.485	ESCHERICHIA COLI	CYTOSOLIC PROTEIN
5GNB	A	L-1N	476	567	165	165	0.866	1.256	1.410	0.019	0.093	-0.670	-0.115	0.485	HUMAN CORONAVIRUS	VIRAL PROTEIN
3G7N	B	L+1N	25	254	24	24	1.205	1.350	1.450	0.003	0.092	-0.318	0.043	0.485	PENICILLIUM EXPANSUM	HYDROLASE
1AK0	A	L+1C	72	217	71	71	1.237	1.428	1.539	0.004	0.092	-0.446	-0.027	0.486	PENICILLIUM CITRINUM	ENDONUCLEASE

Continued on next page

Table S6 – *Continued from previous page*

code	chain	type	open	close	tail1	tail2	a	b	c	Asph	Pred_A	Prolat	Pred_P	motif_P	org	func
1AST	A	L+1N	42	198	41	41	1.067	1.349	1.470	0.008	0.092	-0.628	-0.014	0.486	ASTACUS ASTACUS	HYDROLASE(METALLOPROTEINASE)
4JWO	A	L-1N	154	263	99	99	1.030	1.082	1.139	0.001	0.093	0.095	-0.078	0.486	PLANCTOMYCES LIMNOPHILUS	PHOSPHATE BINDING PROTEIN
5H4U	A	L+1N	88	203	87	87	1.010	1.248	1.296	0.006	0.093	-0.892	-0.068	0.487	CRYPTOPYGUS ANTARCTICUS	HYDROLASE
3H5C	B	L+1C	131	233	82	82	0.940	1.247	1.295	0.009	0.093	-0.928	-0.092	0.488	HOMO SAPIENS	HYDROLASE INHIBITOR
3LQB	A	L+1N	50	199	48	48	1.039	1.334	1.467	0.010	0.092	-0.604	-0.022	0.489	DANIO RERIO	HYDROLASE
3A56	A	L-1N	190	286	172	172	1.150	1.221	1.342	0.002	0.093	0.421	-0.104	0.491	CHRYSEOBACTERIUM PROTEOLYTICUM	HYDROLASE
3CTK	A	L+1C	32	212	31	31	1.149	1.363	1.530	0.007	0.092	-0.209	0.008	0.492	BOUGAINVILLEA SPECTABILIS	HYDROLASE
2YK0	A	L-1N	534	614	515	515	0.753	1.698	1.767	0.054	0.093	-0.984	-0.143	0.492	PLASMODIUM FALCIPARUM	MEMBRANE PROTEIN
6F74	A	L-1C	194	278	167	167	0.915	1.102	1.185	0.006	0.093	-0.608	-0.132	0.493	MYCELIOPHTHORA THERMOPHILA	FLAVOPROTEIN
2WTA	A	L+1N	45	214	42	42	1.083	1.382	1.427	0.007	0.092	-0.938	-0.001	0.494	ACINETOBACTER BAUMANNII	HYDROLASE
1QCX	A	L-1N	72	206	71	71	0.951	1.322	1.439	0.014	0.093	-0.766	-0.040	0.495	ASPERGILLUS NIGER	LYASE
3KLJ	A	L+1N	26	286	24	24	1.198	1.814	1.885	0.018	0.092	-0.960	0.060	0.495	CLOSTRIDIUM ACETOBUTYLICUM	OXIDOREDUCTASE
5Y32	B	L+1C	31	126	0	0	0.896	1.169	1.228	0.009	0.093	-0.876	-0.106	0.498	MUS MUSCULUS	IMMUNE SYSTEM
3WA1	A	LL-1,-1	67	161	48	48	1.042	1.147	1.191	0.002	0.096	-0.629	-0.231	0.499	LYSINIBACILLUS SPHAERICUS	TOXIN
2PE4	A	L+1N	43	333	23	23	1.405	1.662	1.786	0.005	0.092	-0.561	0.074	0.500	HOMO SAPIENS	HYDROLASE
4JD0	A	L+1N	32	241	35	35	1.227	1.312	1.438	0.002	0.092	0.324	0.031	0.503	THERMOTOGA MARITIMA	TRANSFERASE
1Q3X	A	L-1C	434	552	72	72	0.944	1.214	1.265	0.008	0.093	-0.903	-0.063	0.504	HOMO SAPIENS	HYDROLASE
5J81	A	L-1N	761	894	95	95	0.769	2.837	2.860	0.103	0.093	-1.000	-0.042	0.504	PUUMALA VIRUS	VIRAL PROTEIN
5C9I	A	L-1C	49	138	39	39	1.099	1.138	1.461	0.009	0.093	0.956	-0.120	0.504	ACIDOVORAX SP. MR-S7	HYDROLASE
5X4S	A	L-1C	19	133	1	1	1.126	1.483	1.609	0.011	0.093	-0.724	-0.070	0.505	HUMAN SARS CORONAVIRUS	VIRAL PROTEIN
5J81	A	L-1N	790	913	124	124	0.758	2.519	2.558	0.093	0.093	-0.998	-0.056	0.505	PUUMALA VIRUS	VIRAL PROTEIN
5J81	A	L-1N	775	905	109	109	0.774	2.724	2.751	0.099	0.093	-0.999	-0.046	0.506	PUUMALA VIRUS	VIRAL PROTEIN
4CMR	A	L+1N	193	303	192	192	0.956	1.332	1.468	0.015	0.093	-0.712	-0.077	0.508	PYROCOCCUS SP. ST04	HYDROLASE
1NF2	A	L+1N	35	265	34	34	1.139	1.673	1.712	0.015	0.092	-0.984	0.044	0.509	THERMOTOGA MARITIMA	UNKNOWN FUNCTION
5WRR	A	L-1C	209	319	120	120	1.124	1.280	1.473	0.006	0.093	0.179	-0.077	0.510	HOMO SAPIENS	TRANSFERASE
5WRR	A	L-1C	211	323	122	122	1.110	1.273	1.477	0.007	0.093	0.187	-0.073	0.511	HOMO SAPIENS	TRANSFERASE
5J1T	B	L+1N	310	468	74	74	1.185	1.254	1.451	0.004	0.092	0.729	-0.012	0.512	HOMO SAPIENS	HYDROLASE
4FDI	A	L-1N	308	419	279	279	0.950	1.545	1.616	0.024	0.093	-0.957	-0.075	0.517	HOMO SAPIENS	HYDROLASE
1NPE	A	L-1N	957	1175	44	44	1.215	1.345	1.428	0.002	0.092	-0.369	0.036	0.519	MUS MUSCULUS	STRUCTURAL PROTEIN
2PMV	B	L+1C	8	228	1	1	1.275	1.435	1.649	0.006	0.092	0.244	0.038	0.521	HOMO SAPIENS	TRANSPORT PROTEIN
2CKS	A	L+1N	166	406	40	40	1.299	1.326	1.513	0.002	0.092	0.941	0.050	0.522	THERMOBIFIDA FUSCA	HYDROLASE
4H14	A	L-1C	21	165	6	6	1.317	1.585	1.688	0.005	0.092	-0.686	-0.028	0.523	BOVINE CORONAVIRUS	VIRAL PROTEIN
6BSU	A	L+1N	162	384	46	46	1.268	1.473	1.485	0.003	0.092	-0.989	0.039	0.523	ARABIDOPSIS THALIANA	TRANSFERASE
2D5W	A	L+1C	314	458	313	313	1.174	1.373	1.477	0.004	0.092	-0.507	-0.028	0.525	THERMUS THERMOPHILUS	PEPTIDE BINDING PROTEIN
3ZPX	A	L+1N	101	273	87	87	1.056	1.495	1.574	0.014	0.092	-0.911	0.001	0.525	USTILAGO MAYDIS	HYDROLASE
5XWQ	A	LL+1,-1	192	349	115	115	1.047	1.636	1.694	0.020	0.093	-0.970	-0.105	0.525	RHIZOMUCOR MIEHEI	HYDROLASE
3BU7	A	L-1C	100	323	81	81	1.240	1.542	1.585	0.006	0.092	-0.941	0.040	0.527	SILICIBACTER POMEROYI	OXIDOREDUCTASE
4MMU	B	L-1C	155	290	18	18	1.120	1.575	1.659	0.013	0.093	-0.906	-0.039	0.531	HOMO SAPIENS	VIRAL PROTEIN
3DJL	A	L-1N	28	540	25	25	1.532	1.975	2.147	0.009	0.092	-0.681	0.135	0.534	ESCHERICHIA COLI	OXIDOREDUCTASE
4HJ1	A	L-1N	771	965	83	83	0.848	2.879	2.906	0.095	0.092	-0.999	0.020	0.534	RIFT VALLEY FEVER VIRUS	VIRAL PROTEIN
3U4Y	A	L-1N	48	319	44	44	1.308	1.395	1.566	0.003	0.092	0.531	0.066	0.536	DESULFOTOMACULUM ACETOXIDANS	UNKNOWN FUNCTION
1CPY	A	L+1N	56	298	55	55	1.364	1.699	1.875	0.008	0.092	-0.504	0.051	0.538	SACCHAROMYCES CEREVISIAE	HYDROLASE (CARBOXYPEPTIDASE)
1OT5	A	LL+1,-1	230	377	107	107	1.156	1.289	1.450	0.004	0.093	0.171	-0.119	0.539	SACCHAROMYCES CEREVISIAE	HYDROLASE
5BO6	A	L+1N	176	379	86	86	1.218	1.505	1.567	0.006	0.092	-0.877	0.026	0.539	HOMO SAPIENS	TRANSFERASE
4N03	A	L+1N	89	347	54	54	1.216	1.688	1.768	0.012	0.092	-0.920	0.059	0.542	THERMOMONOSPORA CURVATA	TRANSPORT PROTEIN
4KK7	A	L-1C	150	345	76	76	0.992	1.839	1.890	0.034	0.092	-0.989	0.020	0.545	MYCOBACTERIUM TUBERCULOSIS	PROTEIN TRANSPORT
4KP1	A	L+1C	102	365	100	100	1.301	1.568	1.701	0.006	0.092	-0.542	0.062	0.547	METHANOCALDOCOCCUS JANNASCHII	ISOMERASE
1NSC	A	L-1C	86	419	10	10	1.418	1.586	1.640	0.002	0.092	-0.765	0.090	0.552	INFLUENZA B VIRUS	HYDROLASE(O-GLYCOSYL)
4CPN	A	L-1C	86	419	10	10	1.414	1.583	1.638	0.002	0.092	-0.753	0.090	0.552	INFLUENZA B VIRUS	HYDROLASE
5XMG	A	L-1C	29	222	3	3	1.086	1.903	1.957	0.029	0.092	-0.986	0.019	0.553	PSEUDOMONAS AERUGINOSA	IMMUNE SYSTEM
4B7Q	A	L-1C	92	417	9	9	1.398	1.545	1.602	0.002	0.092	-0.675	0.088	0.556	INFLUENZA A VIRUS	HYDROLASE
1AC5	A	L+1N	79	345	78	78	1.438	1.772	1.976	0.008	0.092	-0.404	0.063	0.558	SACCHAROMYCES CEREVISIAE	CARBOXYPEPTIDASE

Continued on next page

Table S6 – *Continued from previous page*

code	chain	type	open	close	tail1	tail2	a	b	c	Asph	Pred_A	Prolat	Pred_P	motif_P	org	func
4KNC	B	L-1C	44	229	2	2	1.188	1.608	1.747	0.012	0.092	-0.751	0.013	0.561	PSEUDOMONAS AERUGINOSA	SUGAR BINDING PROTEIN
2YHG	A	LL+1,-1	564	779	41	41	1.256	1.536	1.580	0.005	0.091	-0.929	-0.041	0.563	SACCHAROPHAGUS DEGRADANS	HYDROLASE
6H7D	A	L+1N	77	449	56	56	1.365	1.904	2.026	0.013	0.092	-0.867	0.103	0.567	ARABIDOPSIS THALIANA	MEMBRANE PROTEIN
2BB6	A	L+1C	3	252	2	2	1.354	1.501	1.747	0.006	0.092	0.425	0.055	0.576	BOS TAURUS	TRANSPORT PROTEIN
1E4M	M	L+1C	6	438	3	3	1.624	1.695	1.855	0.002	0.092	0.613	0.119	0.578	SINAPIS ALBA	HYDROLASE
1E4M	M	L+1C	14	434	11	11	1.598	1.709	1.833	0.002	0.092	0.097	0.116	0.579	SINAPIS ALBA	HYDROLASE
2E1V	A	L-1N	125	433	119	119	1.522	1.736	1.811	0.003	0.092	-0.724	0.081	0.582	CHRYSANTHEMUM X MORIFOLIUM	TRANSFERASE
4H0W	A	L-1N	418	637	417	417	1.211	1.578	1.608	0.008	0.092	-0.979	0.037	0.585	HOMO SAPIENS	METAL TRANSPORT
5K9H	A	L-1C	6	424	2	2	1.328	2.014	2.147	0.019	0.092	-0.897	0.115	0.602	UNIDENTIFIED	HYDROLASE
4H0W	A	L+1N	402	674	401	401	1.313	1.552	1.667	0.005	0.092	-0.565	0.066	0.602	HOMO SAPIENS	METAL TRANSPORT

S4.4 List of proteins structures used

11BA, 12E8, 132L, 152L, 153L, 1A0H, 1A0M, 1A1M, 1A1P, 1A2Q, 1A2V, 1A39, 1A3D, 1A3P, 1A43, 1A4A, 1A4R, 1A67, 1A6A, 1A75, 1A7F, 1A8D, 1A8M, 1A8O, 1A99, 1A9V, 1AAL, 1AB1, 1AB9, 1ABR, 1ABT, 1AC0, 1AC5, 1ACJ, 1ACW, 1ADX, 1ADZ, 1AF6, 1AFA, 1AFH, 1AG7, 1AGG, 1AGQ, 1AGT, 1AGY, 1AIR, 1AIS, 1AIW, 1AJ1, 1AJB, 1AJJ, 1AJK, 1AJO, 1AK0, 1AK2, 1AKG, 1AKP, 1AM5, 1AN1, 1ANS, 1AO3, 1AO6, 1AOC, 1AOG, 1AOL, 1APA, 1APF, 1APO, 1APQ, 1APY, 1AQH, 1AR1, 1ARB, 1ARP, 1AS5, 1AST, 1ATJ, 1ATL, 1ATN, 1AU1, 1AUN, 1AUQ, 1AUT, 1AV3, 1AVB, 1AVD, 1AVG, 1AWB, 1AX8, 1AXG, 1AXH, 1AXK, 1AY2, 1AY7, 1AYJ, 1AYO, 1AZ6, 1AZZ, 1B05, 1B09, 1B0P, 1B10, 1B1V, 1B1Z, 1B2M, 1B2P, 1B30, 1B37, 1B44, 1B45, 1B4B, 1B56, 1B6U, 1B7G, 1B8E, 1B8M, 1B8W, 1B90, 1B9G, 1B9W, 1BBG, 1BBI, 1BBN, 1BCC, 1BCK, 1BCP, 1BDS, 1BEG, 1BEI, 1BF2, 1BG8, 1BGK, 1BH4, 1BHE, 1BHT, 1BHU, 1BI6, 1BIH, 1BII, 1BIK, 1BJ1, 1BK1, 1BK7, 1BKC, 1BKU, 1BMO, 1BMP, 1BNE, 1BNL, 1BOE, 1BOL, 1BOM, 1BOQ, 1BOS, 1BOY, 1BP1, 1BP3, 1BQC, 1BQS, 1BQU, 1BR9, 1BRV, 1BRZ, 1BTE, 1BUS, 1BUY, 1BV4, 1BVO, 1BVW, 1BWZ, 1BX7, 1BY3, 1BYF, 1BYH, 1C01, 1C1Z, 1C2A, 1C3D, 1C3Y, 1C4B, 1C4E, 1C5A, 1C5F, 1C5M, 1C6W, 1C7K, 1C7S, 1C9P, 1C9U, 1CBG, 1CCZ, 1CD1, 1CD9, 1CDG, 1CDH, 1CDQ, 1CE3, 1CE4, 1CEF, 1CFB, 1CFE, 1CFH, 1CHL, 1CHP, 1CID, 1CIV, 1CIX, 1CJQ, 1CKL, 1CL7, 1CLV, 1CM9, 1CMR, 1CN4, 1CNL, 1CNN, 1CNS, 1CNV, 1COU, 1CP7, 1CPO, 1CPY, 1CQE, 1CQK, 1CR8, 1CS6, 1CSA, 1CSB, 1CTI, 1CTN, 1CTO, 1CVI, 1CVS, 1CVW, 1CW5, 1CW6, 1CWA, 1CWR, 1CWV, 1CX1, 1CX8, 1CXP, 1CXW, 1CXY, 1CYA, 1CYB, 1CYN, 1CZ1, 1CZ6, 1CZF, 1CZQ, 1CZS, 1D0G, 1D0N, 1D0S, 1D1H, 1D2B, 1D2L, 1D2T, 1D5Q, 1D6B, 1D6G, 1D6R, 1DAN, 1DCT, 1DE3, 1DEC, 1DEO, 1DG2, 1DIL, 1DJ7, 1DKC, 1DKM, 1DL0, 1DL5, 1DMT, 1DN2, 1DOY, 1DP4, 1DPE, 1DQB, 1DQC, 1DQE, 1DQG, 1DQT, 1DR9, 1DSN, 1DT3, 1DTV, 1DU9, 1DVA, 1DW0, 1DX0, 1DX5, 1DY8, 1DYL, 1DYS, 1DZ7, 1DZO, 1E0F, 1E20, 1E3G, 1E3U, 1E4E, 1E4J, 1E4M, 1E5B, 1E5T, 1E5U, 1E74, 1E75, 1E76, 1E88, 1E8C, 1E8E, 1E8P, 1E8R, 1E8T, 1E9L, 1E9T, 1EAJ, 1EAK, 1EB6, 1EBA, 1ECE, 1ECI, 1EDN, 1EDP, 1EEJ, 1EF7, 1EFE, 1EFX, 1EGF, 1EGT, 1EH5, 1EH9, 1EHD, 1EHS, 1EI0, 1EIA, 1EIT, 1EL0, 1EMN, 1EMR, 1EMX, 1EPA, 1EPF, 1EQF, 1ERC, 1ERD, 1ERM, 1ERY, 1ES7, 1ESC, 1ESL, 1ETE, 1ETH, 1ETL, 1ETM, 1ETN, 1EUT, 1EWS, 1EWW, 1EX1, 1EX2, 1EXG, 1EXT, 1EXZ, 1EZG, 1EZM, 1EZV, 1F02, 1F0N, 1F2G, 1F3K, 1F53, 1F56, 1F5M, 1F5Y, 1F7E, 1F88, 1F8R, 1F8S, 1F9P, 1FAO, 1FAZ, 1FBL, 1FBR, 1FC7, 1FCD, 1FCU, 1FD3, 1FEL, 1FG9, 1FGD, 1FGE, 1FGP, 1FGX, 1FH3, 1FI2, 1FIB, 1FJG, 1FJN, 1FJR, 1FL7, 1FLC, 1FLE, 1FLT, 1FOA, 1FOB, 1FPR, 1FRF, 1FSI, 1FU3, 1FUL, 1FUV, 1FVL, 1FWO, 1FWY, 1FYB, 1FYV, 1FZA, 1FZD, 1FZG, 1G0Y, 1G12, 1G13, 1G1C, 1G1P, 1G26, 1G2G, 1G40, 1G44, 1G66, 1G6E, 1G72, 1G84, 1G8J, 1G8Q, 1G8T, 1G9I, 1G9P, 1GA1, 1GA3, 1GAK, 1GAX, 1GEN, 1GGE, 1GIB, 1GJE, 1GK8, 1GKA, 1GL0, 1GL4, 1GM2, 1GNA, 1GNB, 1GNY, 1GOF, 1GP0, 1GP7, 1GPQ, 1GQB, 1GTP, 1GU3, 1GV8, 1GWB, 1GXS, 1GXY, 1GZJ, 1H0B, 1H0H, 1H0Z, 1H12, 1H20, 1H59, 1H8P, 1H8V, 1H9H, 1HA8, 1HA9, 1HAE, 1HCC, 1HCF, 1HCN, 1HD9, 1HDL, 1HE7, 1HEV, 1HI7, 1HIA, 1HIC, 1HJE, 1HJZ, 1HKF, 1HKY, 1HLG, 1HM6, 1HM7, 1HML, 1HN6, 1HO3, 1HO5, 1HP3, 1HP4, 1HP8, 1HP9, 1HPJ, 1HQQ, 1HRT, 1HSL, 1HTN, 1HTY, 1HV6, 1HVW, 1HVZ, 1HX1, 1HX2, 1HXL, 1HXN, 1HXZ, 1HY2, 1HY9, 1HYK, 1HYP, 1I17, 1I1J, 1I1R, 1I25, 1I26, 1I2U, 1I39, 1I5P, 1I6A, 1I6Y, 1I85, 1I8E, 1I8N, 1I93, 1I98, 1I9B, 1IAM, 1IAR, 1IC9, 1ICA, 1ICF, 1IEN, 1IEO, 1IHN, 1IIY, 1IJ9, 1IJV, 1IJX, 1IJY, 1IKF, 1IKO, 1IL6, 1ILE, 1ILK, 1ILR, 1IM1, 1IM3, 1IM7, 1IM9, 1IMI, 1IMT, 1IMW, 1IOD, 1IOO, 1IRL, 1IRU, 1ISF, 1ISV, 1ITU, 1ITV, 1IV8, 1IVH, 1IVO, 1IW4, 1IWO, 1IXT, 1IXU, 1IYC, 1J0T, 1J1T, 1J2E, 1J2F, 1J3E, 1J7M, 1J7V, 1J8F, 1J8I, 1J8R, 1JB0, 1JBF, 1JBI, 1JBJ, 1JBL, 1JBN, 1JBU, 1JC9, 1JCM, 1JDA, 1JDP, 1JER, 1JFM, 1JFN, 1JFR, 1JFU, 1JFV, 1JFX, 1JI8, 1JIW, 1JJU, 1JJZ, 1JK4, 1JLI, 1JLZ, 1JM1, 1JMA, 1JMC, 1JOV, 1JPE, 1JPY, 1JQR, 1JR8, 1JRF, 1JRH, 1JS4, 1JTA, 1JTG, 1JU2, 1JU8, 1JWF, 1JXC, 1K12, 1K19, 1K36, 1K3B, 1K64, 1K7T, 1K9A, 1KAL, 1KAT, 1KB7, 1KB8, 1KBE, 1KCN, 1KCO, 1KCQ, 1KD7, 1KDG, 1KDV, 1KDY, 1KDZ, 1KE1, 1KE2, 1KFP, 1KG0, 1KG1, 1KGY, 1KI0, 1KIU, 1KJ6, 1KKH, 1KKO, 1KKT, 1KLO, 1KLX, 1KMA, 1KMX, 1KNL, 1KP6, 1KPT, 1KQH, 1KS0, 1KS6, 1KS8, 1KS9, 1KSI, 1KSQ, 1KT6, 1KTB, 1KVD, 1KVF, 1KVG, 1KW2, 1KWD, 1KWE, 1KWN, 1KZQ, 1L1D, 1L1L, 1L2Q, 1L3E, 1L3W, 1L3Y, 1L6H, 1L6P, 1L6U, 1L6X, 1L6Z, 1L8J, 1L9L, 1LB7, 1LBS, 1LBU, 1LDJ, 1LDL, 1LE5, 1LE6, 1LFC, 1LG4, 1LK9, 1LKI, 1LMM, 1LN1, 1LNL, 1LQL, 1LQV, 1LR5, 1LRW, 1LSH, 1LSL, 1LSY, 1LYA, 1M12, 1M1G, 1M1J, 1M2C, 1M3A, 1M4E, 1M4F, 1M4L, 1M4M, 1M63, 1M8A, 1MA2, 1MA5, 1MF7, 1MF8, 1MG1, 1MG2, 1MHD, 1MII, 1MIK, 1MJN, 1MKC, 1MKF, 1MKN, 1MM0, 1MMC, 1MO9, 1MOF, 1MPV, 1MPZ, 1MQ8, 1MR0, 1MT3, 1MTQ, 1MVZ, 1MWO, 1MWP, 1MXN, 1MXP, 1MYN, 1N09, 1N0A, 1N0C, 1N0D, 1N0L, 1N1U, 1N26, 1N2Y, 1N33, 1N5H, 1N69, 1N6U, 1N7D, 1N7U, 1N7Z, 1N9E, 1NBQ, 1NBW, 1NC8, 1NE5, 1NEP, 1NF2, 1NFU, 1NIK, 1NIL, 1NIM, 1NKG, 1NKL, 1NLT, 1NOT, 1NOY, 1NPE, 1NPO, 1NPU, 1NSC, 1NXN, 1NY7, 1NYK, 1NYO, 1NZI, 1O4W, 1O5E, 1O5I, 1O63, 1O7B, 1O7V, 1O86, 1O8R, 1O9V, 1OBR, 1OC0, 1OCC, 1OD7, 1OEE, 1OF9, 1OGM, 1OGQ, 1OGS, 1OH1, 1OHT, 1OIG, 1OJV, 1OK0, 1OKJ, 1OM3, 1OMC, 1OOF, 1OP3, 1OP4, 1OP5, 1OQC, 1OQD, 1OQE, 1OQV, 1OQW, 1ORG, 1ORX, 1OSG, 1OSX, 1OT5, 1OUO, 1OUV, 1OWT, 1OX1, 1P1P, 1P3C, 1P6F, 1P6S, 1P9G, 1P9M, 1PAJ, 1PAK, 1PAN, 1PAO, 1PB5, 1PCE, 1PCM, 1PDK, 1PEN, 1PEU, 1PG1, 1PGS, 1PJA, 1PJU, 1PJV, 1PJW, 1PK6, 1PKO, 1PLR, 1PM4, 1PMX, 1PNB, 1POC, 1POZ, 1PP5, 1PQR, 1PSR, 1PSY, 1PVZ, 1PXZ, 1PZS, 1Q01, 1Q25, 1Q2J, 1Q35, 1Q3M, 1Q3X, 1Q47, 1Q55, 1Q5F, 1Q77, 1Q7M, 1Q90, 1Q98, 1QAI, 1QB5, 1QCX, 1QDP, 1QFB, 1QFT, 1QFX, 1QGI, 1QH2, 1QI9, 1QJV, 1QK7, 1QLW, 1QMW, 1QNG, 1QNH, 1QNO, 1QNX, 1QO6, 1QR3, 1QS3, 1QSA, 1QU0, 1QUP, 1QVK, 1QVL, 1QX9, 1R02, 1R0B, 1R1M, 1R1Z, 1R2M, 1R5Z, 1R8O, 1R8T, 1R9I, 1R9L, 1REW, 1RFX, 1RGX, 1RHF, 1RID, 1RIE, 1RJ1, 1RJH, 1RJI, 1RJT, 1RK4, 1RK7, 1RKI, 1RKK, 1RLY, 1RMG, 1RMK, 1RMR, 1RNI, 1ROF, 1RPB, 1RR9, 1RU4, 1RW5, 1RXD, 1RXL, 1RY7, 1S2J, 1S3A, 1S4N, 1S4Q, 1S62, 1S6D, 1S6W, 1S7D, 1S7P, 1S8K, 1SFI, 1SFO, 1SFP, 1SFS, 1SGL, 1SHI, 1SKI, 1SKK, 1SKL, 1SKZ, 1SLA, 1SLD, 1SLE, 1SM7, 1SML, 1SMO, 1SMV, 1SOC, 1SOP, 1SR4, 1SRA, 1SRZ, 1SS3, 1SSL, 1SSZ, 1ST8, 1SUM, 1SVB, 1SVQ, 1SXR, 1SZH, 1SZL, 1SZN, 1T0P, 1T0W, 1T34, 1T50, 1T61, 1T7H, 1T7L, 1T8T, 1T9E, 1TAP, 1TBQ, 1TDW, 1TEF, 1TER, 1TG7, 1TGO, 1TIH, 1TJY, 1TK2, 1TLE, 1TMC, 1TML, 1TMR, 1TOZ, 1TPG, 1TPM, 1TPS, 1TUJ, 1TV0, 1TWA, 1TYE, 1TYO, 1TZQ, 1U10, 1U34, 1U3D, 1U5M, 1U5Y, 1U79, 1UAI, 1UAP, 1UDK, 1UEO, 1UGL, 1UGQ, 1UHG, 1UL2, 1UMZ, 1UOY, 1UPS, 1URK, 1USG, 1UT3,

1UTE, 1UUZ, 1UVF, 1UVG, 1UWF, 1UYA, 1UYB, 1V46, 1V5A, 1V6C, 1V73, 1V7M, 1VB2, 1VB5, 1VBW, 1VCL, 1VCV, 1VE6, 1VF5, 1VFI, 1VHM, 1VIB, 1VKW, 1VLW, 1VMO, 1VPP, 1VSG, 1VWB, 1VWC, 1VWD, 1VWE, 1VWF, 1VWM, 1VWN, 1VWO, 1VWP, 1VZG, 1VZM, 1W07, 1W18, 1W1N, 1W2Q, 1W37, 1W94, 1WBA, 1WC2, 1WCT, 1WD3, 1WDE, 1WDY, 1WFR, 1WFX, 1WG1, 1WHE, 1WIO, 1WK1, 1WKT, 1WKV, 1WLT, 1WO0, 1WO1, 1WO9, 1WOL, 1WQ1, 1WQB, 1WQC, 1WQE, 1WQJ, 1WQK, 1WQS, 1WS8, 1WSC, 1WUZ, 1WVU, 1WWC, 1WWL, 1WYJ, 1X23, 1X3K, 1X3X, 1X42, 1X5V, 1X7K, 1X9D, 1X9F, 1X9R, 1XAR, 1XAU, 1XBH, 1XCD, 1XDT, 1XFE, 1XGA, 1XGB, 1XGC, 1XHH, 1XHN, 1XI7, 1XIW, 1XJT, 1XJU, 1XKF, 1XM7, 1XQ7, 1XQO, 1XSF, 1XT5, 1XTA, 1XU1, 1XU2, 1XU6, 1XV3, 1XVL, 1XWD, 1XWE, 1XX1, 1XXZ, 1XY4, 1XY5, 1XY6, 1XY8, 1XY9, 1XYH, 1Y1B, 1Y43, 1Y49, 1Y4J, 1Y4M, 1Y58, 1Y6W, 1Y7J, 1Y8E, 1Y9L, 1YC0, 1YF4, 1YK1, 1YL8, 1YL9, 1YNT, 1YO8, 1YP8, 1YPY, 1YQT, 1YSH, 1YT6, 1YTD, 1YU6, 1YUK, 1Z1Y, 1Z2G, 1Z2K, 1Z6O, 1Z6V, 1Z8Y, 1Z9M, 1ZAQ, 1ZD0, 1ZE1, 1ZE3, 1ZH1, 1ZJ8, 1ZJK, 1ZKR, 1ZLC, 1ZLS, 1ZLU, 1ZLV, 1ZLW, 1ZMM, 1ZOX, 1ZPX, 1ZT2, 1ZT3, 1ZTY, 1ZWT, 1ZY6, 1ZYB, 2A05, 2A0Z, 2A2B, 2A6V, 2A75, 2A7R, 2A96, 2A9X, 2AB9, 2AGL, 2AJW, 2AO2, 2AP2, 2APO, 2ARP, 2ASK, 2ATG, 2AW2, 2AXI, 2B0U, 2B34, 2B5B, 2B5E, 2B5I, 2B5K, 2B5P, 2B5Q, 2BAF, 2BB6, 2BC7, 2BEM, 2BEY, 2BIC, 2BIS, 2BJO, 2BJU, 2BO2, 2BR8, 2BSK, 2BT1, 2BVB, 2BYP, 2C4B, 2C8S, 2C9A, 2C9T, 2CC0, 2CCV, 2CD9, 2CE0, 2CFS, 2CGJ, 2CK0, 2CK5, 2CKI, 2CKN, 2CKS, 2CL2, 2CLB, 2CMY, 2CMZ, 2COV, 2CQ7, 2CQ9, 2CQW, 2CSB, 2CV3, 2CV4, 2CVB, 2CZQ, 2D1G, 2D3J, 2D3Z, 2D49, 2D56, 2D5N, 2D5W, 2D74, 2D7I, 2D80, 2DCO, 2DDE, 2DDR, 2DDX, 2DE0, 2DET, 2DHO, 2DN6, 2DOH, 2DQA, 2DRU, 2DS2, 2DSP, 2DV2, 2DVZ, 2DW0, 2DX0, 2E0I, 2E1P, 2E1V, 2E1X, 2E21, 2E2F, 2E2N, 2E2S, 2E56, 2E5E, 2E8C, 2E9W, 2EA1, 2EAB, 2EBF, 2EBO, 2EC2, 2EC3, 2EC7, 2ECE, 2EEM, 2EFZ, 2EH6, 2EHF, 2EHG, 2EKC, 2ENG, 2ENS, 2EO9, 2EQQ, 2ERB, 2ERF, 2ERJ, 2ERS, 2ERW, 2ESL, 2ETC, 2EW4, 2EX2, 2F09, 2F2F, 2F2G, 2F3L, 2F3M, 2F4M, 2FAW, 2FBO, 2FDB, 2FEB, 2FFU, 2FHB, 2FHW, 2FLG, 2FMC, 2FN2, 2FP8, 2FQA, 2FQC, 2FR9, 2FRB, 2FUG, 2FUI, 2FX5, 2FXJ, 2G6U, 2G6Z, 2G84, 2G9L, 2GA6, 2GBB, 2GCZ, 2GER, 2GGO, 2GH0, 2GHW, 2GIY, 2GJ7, 2GLU, 2GM7, 2GMF, 2GR9, 2GRK, 2GRU, 2GSN, 2GTL, 2GV1, 2GVD, 2GVU, 2GWC, 2GX1, 2GY5, 2H2R, 2H32, 2H3N, 2H5F, 2H6O, 2H7T, 2H8B, 2H8S, 2H8V, 2H9E, 2H9U, 2HB0, 2HBT, 2HCC, 2HCZ, 2HDL, 2HEV, 2HF9, 2HFG, 2HGF, 2HGO, 2HHI, 2HJ3, 2HLG, 2HLQ, 2HM3, 2HNU, 2HQ4, 2HQY, 2HRE, 2HVV, 2HYE, 2HZB, 2HZQ, 2I1P, 2I28, 2I71, 2IA4, 2IBP, 2IC1, 2ICC, 2ICE, 2ID5, 2IEC, 2IF7, 2IFG, 2IFI, 2IFJ, 2IFR, 2IFZ, 2IGS, 2IGU, 2IH6, 2IH7, 2IHA, 2IKD, 2IKE, 2IM9, 2IPL, 2IPP, 2ISI, 2IW0, 2IXG, 2IY9, 2J15, 2J2Z, 2J4M, 2J4T, 2J4U, 2J4W, 2J5H, 2J5L, 2J6D, 2J85, 2J8J, 2JA4, 2JBX, 2JD4, 2JFG, 2JG0, 2JGW, 2JH1, 2JIG, 2JJS, 2JLP, 2JM2, 2JMI, 2JMR, 2JNI, 2JNR, 2JOB, 2JOD, 2JON, 2JOP, 2JOR, 2JP0, 2JP6, 2JQ8, 2JQW, 2JR3, 2JRW, 2JRY, 2JS7, 2JS9, 2JTK, 2JTO, 2JTU, 2JTY, 2JUQ, 2JUR, 2JUS, 2JUT, 2JUY, 2JV7, 2JVE, 2JVU, 2JWG, 2JWH, 2JX9, 2JYE, 2JYP, 2K10, 2K13, 2K1V, 2K2S, 2K2Y, 2K2Z, 2K35, 2K3G, 2K3J, 2K4R, 2K72, 2K73, 2K8P, 2KAA, 2KB8, 2KB9, 2KBX, 2KC6, 2KD3, 2KDQ, 2KDS, 2KER, 2KGU, 2KGW, 2KIE, 2KIZ, 2KJF, 2KJI, 2KJX, 2KK2, 2KKY, 2KL7, 2KMS, 2KMZ, 2KN0, 2KNJ, 2KNP, 2KOZ, 2KPY, 2KQA, 2KR1, 2KS9, 2KSK, 2KSL, 2KSW, 2KT6, 2KTC, 2KTN, 2KTS, 2KUM, 2KUS, 2KUY, 2KVD, 2KVX, 2KX5, 2KXI, 2KYJ, 2L03, 2L07, 2L1Q, 2L26, 2L27, 2L2R, 2L37, 2L3I, 2L3O, 2L4N, 2L5P, 2L5S, 2L7J, 2L7S, 2L8Y, 2L9H, 2L9X, 2LA0, 2LA7, 2LAQ, 2LBZ, 2LC6, 2LCR, 2LCY, 2LDE, 2LDF, 2LER, 2LG4, 2LG6, 2LGQ, 2LHT, 2LI3, 2LIX, 2LIY, 2LJ9, 2LJM, 2LL3, 2LLR, 2LMK, 2LMZ, 2LN8, 2LOE, 2LPN, 2LQA, 2LQL, 2LQT, 2LQU, 2LQV, 2LQX, 2LR9, 2LRD, 2LRM, 2LS1, 2LSH, 2LSQ, 2LT5, 2LTI, 2LU6, 2LUR, 2LUT, 2LVF, 2LVX, 2LW6, 2LWB, 2LWL, 2LWQ, 2LWS, 2LWT, 2LWU, 2LWV, 2LX6, 2LXG, 2LYE, 2LYF, 2LZ5, 2LZI, 2LZO, 2M05, 2M0P, 2M1P, 2M25, 2M2F, 2M2G, 2M2H, 2M2Q, 2M2S, 2M2X, 2M2Y, 2M35, 2M36, 2M37, 2M3I, 2M3K, 2M3N, 2M45, 2M5G, 2M5X, 2M61, 2M62, 2M6A, 2M6C, 2M6D, 2M6E, 2M6F, 2M6G, 2M6H, 2M6J, 2M74, 2M75, 2M77, 2M78, 2M79, 2M7I, 2M7J, 2M7Z, 2M86, 2M8F, 2M8V, 2MAH, 2MAI, 2MBD, 2MBL, 2MC2, 2MCF, 2MCR, 2MD0, 2MD6, 2MD7, 2MDB, 2MDL, 2MDN, 2MDQ, 2MF3, 2MFJ, 2MFK, 2MFP, 2MFS, 2MFV, 2MFX, 2MFY, 2MG6, 2MG9, 2MGO, 2MGS, 2MH1, 2MH5, 2MHP, 2MHV, 2MHY, 2MI1, 2MI5, 2MIJ, 2MIQ, 2MIT, 2MIX, 2MIZ, 2MJK, 2MK0, 2ML7, 2MLJ, 2MM0, 2MM2, 2MMH, 2MN3, 2MN5, 2MOP, 2MP1, 2MP8, 2MPO, 2MQU, 2MSF, 2MSQ, 2MSX, 2MT8, 2MTM, 2MTO, 2MTT, 2MTU, 2MUH, 2MUN, 2MV1, 2MVA, 2MVO, 2MW3, 2MW7, 2MWR, 2MX8, 2MXQ, 2MYH, 2MYJ, 2MYV, 2MYW, 2MZ0, 2MZ6, 2N17, 2N24, 2N2S, 2N37, 2N4E, 2N4O, 2N59, 2N5C, 2N5K, 2N5Q, 2N5Z, 2N6R, 2N7N, 2N86, 2N8B, 2N8E, 2N8F, 2N8H, 2N8K, 2N99, 2N9Z, 2NAJ, 2NAN, 2NAV, 2NB2, 2NB5, 2NB6, 2NBC, 2NBH, 2NC7, 2NCM, 2ND2, 2ND3, 2NDD, 2NDI, 2NDL, 2NDM, 2NDN, 2NMS, 2NPL, 2NS3, 2NS4, 2NWN, 2NX6, 2NX7, 2O26, 2O4C, 2O5N, 2O5W, 2O9Q, 2O9X, 2OBD, 2ODT, 2ODY, 2OHC, 2OIZ, 2OJU, 2OPD, 2OQ9, 2OQJ, 2OQP, 2OR7, 2ORX, 2OSL, 2OTQ, 2OX2, 2OYA, 2OYK, 2OZ4, 2P23, 2P28, 2P39, 2P3F, 2P3X, 2P4Z, 2P52, 2P5R, 2P9V, 2PA8, 2PE4, 2PET, 2PFC, 2PLF, 2PLX, 2PMV, 2PNE, 2PO8, 2POF, 2POP, 2POS, 2POY, 2PQL, 2PSM, 2PSP, 2PT5, 2PVC, 2Q1M, 2Q3I, 2Q7N, 2Q88, 2Q8T, 2Q9T, 2QHA, 2QJT, 2QKH, 2QKI, 2QKT, 2QLY, 2QPQ, 2QRL, 2QSA, 2QSK, 2QTS, 2QYC, 2QYF, 2QZF, 2R1R, 2R3C, 2R3Z, 2R5B, 2R5D, 2R7E, 2RAL, 2RBG, 2RC7, 2RCK, 2REG, 2RFT, 2RIK, 2RJI, 2RJQ, 2RKN, 2RL8, 2RMA, 2RMB, 2RMC, 2RNG, 2RNL, 2ROO, 2RPJ, 2RPS, 2RPV, 2RQA, 2RSX, 2RTU, 2RTV, 2RTY, 2RU1, 2RU2, 2RUP, 2SAS, 2SIC, 2SOC, 2UUX, 2UWN, 2UZ6, 2V4J, 2V6O, 2V7S, 2VEC, 2VGA, 2VH3, 2VJ2, 2VJ3, 2VN4, 2VQ2, 2VQC, 2VQG, 2VR5, 2VRK, 2VSD, 2VSG, 2VSM, 2VTC, 2VVY, 2VWE, 2VXZ, 2VYO, 2VZB, 2W2M, 2W44, 2W46, 2W57, 2W86, 2W8X, 2WAQ, 2WB7, 2WB9, 2WBF, 2WBT, 2WCY, 2WFJ, 2WFO, 2WFU, 2WFV, 2WG7, 2WGN, 2WGO, 2WII, 2WIN, 2WJ3, 2WMP, 2WNO, 2WR0, 2WTA, 2WV3, 2WVX, 2WX1, 2WY3, 2WYB, 2X11, 2X2C, 2X3H, 2X46, 2X5X, 2X7K, 2X97, 2X9A, 2X9W, 2XC8, 2XCI, 2XET, 2XFB, 2XFC, 2XFD, 2XGG, 2XGL, 2XH8, 2XIO, 2XJH, 2XJI, 2XL7, 2XNK, 2XRB, 2XTJ, 2XU8, 2XV9, 2XVT, 2XXL, 2XYC, 2XZ8, 2Y1B, 2Y1V, 2Y25, 2Y32, 2Y44, 2Y4T, 2Y7B, 2Y7L, 2Y8I, 2Y8S, 2YAU, 2YF1, 2YG2, 2YGQ, 2YGU, 2YHG, 2YIH, 2YIL, 2YK0, 2YOP, 2YOR, 2YQ2, 2YS0, 2YZJ, 2Z0U, 2Z1D, 2Z1K, 2Z3Q, 2Z4I, 2Z4T, 2Z6W, 2Z73, 2Z7E, 2Z7F, 2Z8H, 2ZAG, 2ZG2, 2ZGI, 2ZIY, 2ZJR, 2ZK9, 2ZKI, 2ZOT, 2ZOU, 2ZUM, 2ZX2, 2ZZJ, 3A07, 3A0F, 3A23, 3A2E, 3A2K, 3A56, 3A57, 3A8Y, 3AAP, 3ADA, 3ADR, 3AGN, 3AGR, 3AJA, 3AJF, 3AJV, 3ALP, 3ALZ, 3AP2, 3APQ, 3AQF, 3ASI, 3ATQ, 3AV9, 3AVA, 3AVB, 3AVF, 3AVG, 3AVH, 3AVI, 3AVJ, 3AVK, 3AVL, 3AVM, 3AVN, 3AW9, 3AXS, 3AY2, 3AYR, 3AYY,

3B1J, 3B2D, 3B2M, 3B2U, 3B2Z, 3B43, 3B4V, 3B5H, 3B8F, 3B90, 3BDB, 3BE5, 3BES, 3BFQ, 3BGA, 3BGO, 3BIK, 3BK7, 3BO7, 3BOG, 3BQH, 3BT1, 3BT4, 3BU7, 3BUL, 3BWU, 3BX4, 3C26, 3C59, 3C6E, 3C75, 3C8J, 3C9A, 3CJH, 3CJJ, 3CKM, 3CNP, 3CO5, 3CP7, 3CQO, 3CSL, 3CSY, 3CTK, 3CWY, 3CX5, 3CYS, 3D0J, 3D2D, 3D34, 3D3I, 3D5N, 3DBY, 3DEM, 3DFV, 3DGC, 3DGE, 3DI2, 3DIV, 3DJL, 3DMK, 3DMM, 3DMQ, 3DNF, 3DRX, 3DRZ, 3DTD, 3DV8, 3DWA, 3DXL, 3DXR, 3DZD, 3DZM, 3E1Y, 3E8T, 3ECM, 3EDY, 3EE4, 3EJH, 3EJL, 3ENT, 3EO5, 3EOV, 3EOY, 3EQA, 3EQN, 3ERI, 3ES6, 3EVS, 3EW1, 3F4R, 3F62, 3F6U, 3F83, 3FAC, 3FBY, 3FD4, 3FFK, 3FFV, 3FFZ, 3FGT, 3FHG, 3FIM, 3FJU, 3FLP, 3FMZ, 3FP7, 3FPR, 3FTC, 3FUB, 3FVZ, 3FWZ, 3FX3, 3FZ7, 3FZD, 3G1H, 3G4D, 3G5C, 3G5V, 3G5Y, 3G7M, 3G7N, 3G8B, 3G8L, 3GBY, 3GHG, 3GI7, 3GKH, 3GKK, 3GMF, 3GMI, 3GNU, 3GOV, 3GR5, 3GRH, 3GRW, 3GYX, 3GZT, 3H09, 3H0G, 3H2U, 3H31, 3H3B, 3H3E, 3H49, 3H4W, 3H5C, 3H5V, 3H9V, 3HCJ, 3HCU, 3HDF, 3HEI, 3HHC, 3HJR, 3HKL, 3HN4, 3HOT, 3HWN, 3HYV, 3HZB, 3I08, 3I26, 3I84, 3ICU, 3IEG, 3IJ2, 3IT5, 3IT8, 3IUX, 3IWP, 3IWR, 3IYP, 3IZX, 3J0G, 3J0K, 3J2T, 3J5L, 3J6N, 3J7A, 3J7Z, 3J9F, 3JAD, 3JB4, 3JBC, 3JCU, 3JD6, 3JUU, 3JVF, 3JYH, 3JYZ, 3K1L, 3K3G, 3K3W, 3K48, 3K4P, 3K5Y, 3K65, 3K6S, 3K6Y, 3K7B, 3K94, 3KB6, 3KBQ, 3KD2, 3KDP, 3KFB, 3KG5, 3KGR, 3KIO, 3KLJ, 3KN3, 3KP8, 3KQ0, 3KQ4, 3KS7, 3KTB, 3KTM, 3KVN, 3KWC, 3KXA, 3L0G, 3L0R, 3L35, 3L36, 3L37, 3L47, 3L49, 3L5H, 3L5I, 3LB6, 3LG1, 3LGD, 3LJK, 3LLI, 3LMM, 3LOB, 3LPA, 3LQB, 3LQC, 3LQM, 3LRI, 3LRK, 3LSO, 3LTF, 3LV9, 3LVB, 3LY6, 3LY9, 3M45, 3M5U, 3M61, 3M6K, 3M7O, 3M8T, 3M99, 3MAY, 3ME2, 3MGN, 3MIA, 3MJ6, 3MJG, 3MK7, 3MLS, 3MN8, 3MQL, 3MTR, 3MTW, 3MWQ, 3N08, 3N0W, 3N3O, 3N3R, 3N40, 3N55, 3N7P, 3N90, 3NA6, 3NCM, 3NGG, 3NGW, 3NGY, 3NHV, 3NJW, 3NOK, 3NPE, 3NRF, 3NSJ, 3NSW, 3NT8, 3NTK, 3NVN, 3NVQ, 3NVV, 3O0A, 3O1I, 3O26, 3O3V, 3O44, 3O4O, 3O53, 3O8E, 3O9L, 3OA8, 3ODI, 3ODL, 3OE0, 3OE3, 3OF6, 3OG4, 3OJA, 3OJO, 3ON9, 3OO2, 3OP8, 3OPE, 3OPU, 3OQ3, 3OQU, 3OTK, 3OZH, 3OZP, 3P06, 3P0F, 3P1Z, 3P4P, 3P5C, 3P5S, 3P72, 3P8F, 3PAY, 3PDX, 3PF0, 3PF2, 3PGB, 3PHF, 3PHS, 3PIC, 3PIS, 3PIV, 3PIW, 3PM2, 3PMP, 3PMQ, 3POB, 3PPV, 3PQK, 3PXL, 3Q13, 3Q2U, 3Q3Q, 3Q41, 3Q6K, 3Q6O, 3QDP, 3QE5, 3QF2, 3QHB, 3QL6, 3QNT, 3QQN, 3QSD, 3QT2, 3QTE, 3QTL, 3QTP, 3QV1, 3QVP, 3QW9, 3R08, 3R0L, 3R1V, 3R6B, 3RCQ, 3RDY, 3REZ, 3RFJ, 3RGZ, 3RJV, 3RKP, 3ROI, 3ROJ, 3RRX, 3RT4, 3RZS, 3RZV, 3S0T, 3S35, 3S3Y, 3S4O, 3S63, 3S64, 3S6T, 3S6V, 3S88, 3SFG, 3SH4, 3SHR, 3SI2, 3SM5, 3SOB, 3SOJ, 3SPG, 3SQG, 3SRG, 3SRI, 3SSB, 3SUK, 3SUM, 3SW0, 3T4J, 3T94, 3TBD, 3TC3, 3TCM, 3TGX, 3TNX, 3TQF, 3TRC, 3TVJ, 3TW0, 3U33, 3U3P, 3U3U, 3U4K, 3U4Y, 3UAF, 3UIW, 3ULY, 3UN7, 3UOV, 3USH, 3UTK, 3UUE, 3UXU, 3UYX, 3UZE, 3V0R, 3V39, 3V4P, 3V56, 3V7B, 3VDJ, 3VJP, 3VLA, 3VN5, 3VOC, 3VPB, 3VPI, 3VPJ, 3VRH, 3VTH, 3VUP, 3VX0, 3W0D, 3W14, 3W1E, 3W36, 3W4R, 3W4S, 3W57, 3W5G, 3W81, 3WA1, 3WBN, 3WE7, 3WJM, 3WL5, 3WLS, 3WLT, 3WMP, 3WMT, 3WMZ, 3WNE, 3WNF, 3WNG, 3WNH, 3WOI, 3WP9, 3WUS, 3WX7, 3WXI, 3X0H, 3X2G, 3ZC4, 3ZDF, 3ZFI, 3ZFN, 3ZGC, 3ZH5, 3ZHG, 3ZIB, 3ZK4, 3ZKK, 3ZKT, 3ZLD, 3ZP6, 3ZPM, 3ZPX, 3ZUI, 3ZVQ, 3ZVS, 3ZWZ, 3ZXK, 3ZYP, 3ZYZ, 3ZZO, 4A3X, 4A56, 4A5V, 4A94, 4ABJ, 4AE2, 4AG4, 4AKA, 4AKM, 4AOG, 4AOQ, 4AP5, 4APX, 4AQB, 4ARN, 4ARQ, 4AS2, 4AUP, 4AX2, 4AXV, 4AYB, 4AZ0, 4B0E, 4B1Q, 4B2N, 4B2U, 4B4P, 4B6I, 4B7Q, 4B8V, 4BD9, 4BDW, 4BED, 4BFE, 4BFI, 4BHR, 4BOE, 4BQD, 4BSP, 4BVW, 4BWC, 4C16, 4C53, 4C79, 4C86, 4CC0, 4CCV, 4CD3, 4CDG, 4CGE, 4CHM, 4CII, 4CJM, 4CK4, 4CMR, 4CPN, 4CRY, 4CVB, 4CVW, 4CYF, 4D0J, 4D0T, 4D40, 4D6T, 4D6W, 4D9G, 4DA2, 4DBG, 4DCB, 4DD4, 4DD6, 4DDD, 4DEQ, 4DF0, 4DG6, 4DGC, 4DI6, 4DIP, 4DKC, 4DM5, 4DOH, 4DT5, 4DVK, 4DWN, 4DXR, 4DZ1, 4E0I, 4E40, 4E5X, 4E97, 4E9I, 4E9K, 4EBY, 4ECO, 4EDI, 4EEE, 4EM5, 4EO0, 4EO3, 4EOB, 4EPI, 4EQ7, 4EQA, 4ERS, 4ES7, 4ESQ, 4ETY, 4EVQ, 4EXP, 4EZ1, 4F0J, 4F1J, 4F23, 4F2M, 4F3F, 4F4O, 4F5C, 4F7B, 4FAO, 4FAS, 4FAY, 4FDI, 4FFE, 4FGU, 4FNK, 4FPR, 4FR0, 4FTE, 4FTF, 4FVS, 4FXG, 4FYP, 4G2A, 4G2K, 4G4A, 4G4G, 4G59, 4G7N, 4G7W, 4G7X, 4GBO, 4GDX, 4GE1, 4GEY, 4GFJ, 4GFX, 4GI3, 4GIP, 4GLY, 4GN2, 4GQR, 4GQZ, 4GV5, 4GVU, 4GW1, 4GW5, 4GWN, 4GZ9, 4H04, 4H0W, 4H14, 4H5S, 4HCS, 4HGC, 4HGK, 4HJ1, 4HKJ, 4HMC, 4HN7, 4HQ1, 4HQF, 4HR0, 4HRN, 4HS9, 4HSS, 4HW5, 4HWB, 4HWM, 4HWU, 4HY7, 4HYQ, 4HZC, 4I05, 4I0W, 4I2X, 4I6X, 4IB5, 4IHU, 4IJ3, 4IJD, 4IJJ, 4ILD, 4ILQ, 4IOI, 4IPU, 4IRQ, 4IVG, 4IXP, 4IZJ, 4J30, 4J3Q, 4JD0, 4JD9, 4JF3, 4JFN, 4JGL, 4JHS, 4JHY, 4JJJ, 4JJM, 4JJO, 4JMO, 4JNO, 4JOD, 4JP6, 4JPH, 4JPJ, 4JS1, 4JUR, 4JVU, 4JVY, 4JWO, 4K07, 4K0U, 4K0V, 4K17, 4K1E, 4K3U, 4K6L, 4K7T, 4K82, 4K8Y, 4K90, 4K92, 4K94, 4KAV, 4KBS, 4KEL, 4KG7, 4KK7, 4KM6, 4KNC, 4KP1, 4KQ9, 4KQA, 4KRX, 4KT1, 4KT3, 4KTS, 4KTU, 4KVF, 4KYP, 4L05, 4L3N, 4LB1, 4LE1, 4LI1, 4LL4, 4LLD, 4LLO, 4LLQ, 4LMO, 4LMQ, 4LOS, 4LP5, 4LQ6, 4LSC, 4LU2, 4LV5, 4LV8, 4LVN, 4LVQ, 4LXO, 4M6E, 4MAA, 4MGX, 4MHF, 4MHX, 4MIR, 4MJG, 4MJQ, 4MKM, 4ML1, 4MLZ, 4MM3, 4MMO, 4MMR, 4MMU, 4MNN, 4MQX, 4MS7, 4MSP, 4MX4, 4MXB, 4MXD, 4MXG, 4MXH, 4MXN, 4MZV, 4MZZ, 4N03, 4N06, 4N2Z, 4N30, 4N3T, 4N4N, 4N6M, 4N7C, 4N8N, 4NAG, 4NDS, 4NDZ, 4NI3, 4NKQ, 4NNR, 4NOA, 4NOF, 4NOO, 4NT5, 4NU2, 4NV2, 4NWV, 4NWW, 4NYQ, 4O3U, 4O4A, 4O4O, 4O5J, 4O6K, 4O8J, 4O98, 4ODD, 4OF8, 4OFK, 4OFL, 4OFY, 4OG9, 4OGR, 4OI9, 4OIE, 4OJ7, 4OKV, 4OM7, 4OMB, 4ON3, 4OPB, 4OQ1, 4OW1, 4OW9, 4OWJ, 4OZK, 4OZU, 4OZV, 4P04, 4P27, 4P39, 4P9C, 4PAG, 4PBX, 4PDI, 4PE6, 4PH8, 4PHO, 4PLM, 4PMK, 4PSC, 4PT1, 4PXV, 4Q5Y, 4Q6I, 4Q82, 4Q89, 4Q8K, 4QBQ, 4QCI, 4QDS, 4QGE, 4QI3, 4QI7, 4QJY, 4QNN, 4QS4, 4QTQ, 4QXF, 4QXL, 4R0L, 4R12, 4R1T, 4R2B, 4R38, 4R4X, 4R5Q, 4R72, 4R7Q, 4R9L, 4RCA, 4RDQ, 4RE1, 4RGD, 4RHZ, 4RKU, 4RL4, 4RSU, 4RU0, 4RU1, 4RUN, 4RUQ, 4RUW, 4RWS, 4RYA, 4S0U, 4S3L, 4TJV, 4TMV, 4TOT, 4TPU, 4TPV, 4TRG, 4TTK, 4TTV, 4TUJ, 4TUK, 4TUL, 4TVE, 4TVS, 4TVV, 4TWE, 4U0Q, 4U2W, 4U2X, 4U4B, 4U5B, 4U7D, 4U7L, 4U9V, 4UB9, 4UEY, 4UF7, 4UHZ, 4UI2, 4UIQ, 4UOJ, 4UQF, 4UY8, 4UYS, 4V00, 4V2B, 4V3D, 4W50, 4WCE, 4WFA, 4WFB, 4WJG, 4WK4, 4WL2, 4WM0, 4WMO, 4WMQ, 4WNF, 4WRN, 4WTP, 4WVZ, 4X0J, 4X0N, 4X0W, 4X1N, 4X1P, 4X1Q, 4X1R, 4X1S, 4X4M, 4X63, 4X7G, 4X90, 4X9Z, 4XGO, 4XHJ, 4XHR, 4XIN, 4XIY, 4XIZ, 4XJN, 4XJY, 4XL1, 4XMQ, 4XNP, 4XOJ, 4XP8, 4XSS, 4XT1, 4XT3, 4XTN, 4XUU, 4XVH, 4XVV, 4XWH, 4XZ6, 4Y1T, 4Y4C, 4Y4Y, 4Y61, 4Y7S, 4Y7Y, 4YCG, 4YDV, 4YEB, 4YF4, 4YFA, 4YFC, 4YFD, 4YGF, 4YH7, 4YHQ, 4YIZ, 4YJ0, 4YKI, 4YKK, 4YLN, 4YV9, 4YYF, 4Z05, 4Z09, 4Z0C, 4Z11, 4Z1P, 4Z39, 4Z42, 4Z4A, 4Z80, 4Z8I, 4Z8N, 4Z8W, 4Z9N, 4ZAI, 4ZCE, 4ZEZ, 4ZGV, 4ZHE, 4ZHL, 4ZHM, 4ZHU, 4ZI8, 4ZJ4, 4ZJ5, 4ZJ6, 4ZJD, 4ZJF, 4ZJM, 4ZKN, 4ZKQ, 4ZKS, 4ZRI, 5A07, 5A0E, 5A0G, 5A0L, 5A0N, 5A2F, 5A3L, 5A5X, 5A63, 5A6W, 5A96, 5A9D, 5AC3,

5AEA, 5AEJ, 5ANN, 5AO1, 5AO7, 5AOH, 5APA, 5AQ1, 5ARF, 5AX2, 5AYK, 5AZW, 5AZX, 5B04, 5B0H, 5B3C, 5B4S, 5B5I, 5B5K, 5B5S, 5BJT, 5BMQ, 5BO1, 5BO6, 5BPU, 5BTU, 5BV3, 5BV8, 5BWI, 5BXL, 5C0H, 5C14, 5C1F, 5C67, 5C86, 5C9I, 5CA4, 5CCF, 5CDI, 5CEZ, 5CJH, 5CNZ, 5CO5, 5CR9, 5CTR, 5CWQ, 5CXM, 5CYL, 5CYY, 5CZK, 5CZR, 5D0O, 5D71, 5D8M, 5D9E, 5DAZ, 5DI8, 5DIC, 5DJC, 5DJX, 5DM8, 5DRZ, 5DSS, 5DVK, 5DVL, 5DVM, 5DVN, 5DZE, 5DZW, 5E13, 5E27, 5E2M, 5E5Q, 5E8D, 5E8M, 5E9D, 5EB9, 5EBB, 5EDF, 5EFM, 5EJB, 5EK5, 5EKI, 5EKU, 5ELI, 5EO9, 5EOC, 5ERP, 5ETU, 5EUK, 5EVE, 5EVF, 5EWK, 5EX1, 5EX2, 5F0E, 5F18, 5F1D, 5F1N, 5F44, 5F4H, 5F73, 5F84, 5F88, 5F8T, 5F8X, 5F8Z, 5FDY, 5FF6, 5FFG, 5FGN, 5FGR, 5FHC, 5FI2, 5FI9, 5FID, 5FIG, 5FMV, 5FN6, 5FN7, 5FNR, 5FO9, 5FOB, 5FOJ, 5FOY, 5FP1, 5FR8, 5FRH, 5FTT, 5FUC, 5FV5, 5FWT, 5FYO, 5FZP, 5FZW, 5G1U, 5G23, 5G25, 5G38, 5G6U, 5GGI, 5GJQ, 5GMT, 5GN2, 5GNB, 5GNE, 5GSF, 5GU5, 5GV3, 5GVA, 5GVO, 5GYB, 5GYQ, 5GYY, 5GZA, 5H05, 5H18, 5H1H, 5H1I, 5H1Q, 5H1S, 5H4S, 5H4U, 5H5Q, 5H5R, 5H5S, 5H60, 5H6B, 5H8V, 5H9K, 5HCC, 5HCT, 5HDF, 5HHA, 5HI8, 5HIO, 5HJ3, 5HL7, 5HNW, 5HOW, 5HPT, 5HQN, 5HRM, 5HSF, 5HZ2, 5HZ7, 5I0Z, 5I2I, 5I4H, 5I6A, 5I6C, 5I6X, 5I99, 5IAZ, 5ICZ, 5IEC, 5IHB, 5II0, 5IIA, 5IIP, 5INZ, 5IOJ, 5IOP, 5IPO, 5IR1, 5IRX, 5ITF, 5IU1, 5IUE, 5IV2, 5IVZ, 5IW5, 5IX5, 5IX8, 5IXG, 5IXP, 5IZ3, 5J11, 5J1T, 5J3Z, 5J81, 5J83, 5J89, 5J8T, 5JAK, 5JBT, 5JCD, 5JCE, 5JG9, 5JHI, 5JI4, 5JK5, 5JKP, 5JME, 5JOT, 5JP2, 5JPL, 5JQF, 5JQP, 5JQY, 5JT8, 5JTS, 5JX6, 5JYQ, 5JYS, 5JZH, 5JZU, 5JZZ, 5K0Y, 5K1E, 5K21, 5K6D, 5K6U, 5K6W, 5K9H, 5KBC, 5KC9, 5KCB, 5KDV, 5KEM, 5KEN, 5KI9, 5KKM, 5KLC, 5KLH, 5KN5, 5KNM, 5KP9, 5KUY, 5KVM, 5KVN, 5KWO, 5KWP, 5KWX, 5KWZ, 5KX0, 5KX1, 5KX2, 5KX4, 5KZ5, 5KZP, 5L0Q, 5L0R, 5L1X, 5L34, 5L3Z, 5L73, 5L74, 5L7C, 5L7R, 5L9W, 5LA4, 5LAH, 5LAJ, 5LAL, 5LC2, 5LC5, 5LCS, 5LDX, 5LDY, 5LFR, 5LFZ, 5LGD, 5LGK, 5LHT, 5LJ3, 5LMN, 5LNK, 5LSO, 5LSP, 5LTL, 5LUW, 5LUY, 5LV1, 5LWZ, 5LY9, 5LZL, 5M0K, 5M0R, 5M0W, 5M3F, 5M4T, 5M5E, 5M5Z, 5M8S, 5M9N, 5MAB, 5MDH, 5ME0, 5MFA, 5MJ4, 5MKF, 5MLC, 5MO9, 5MRI, 5MRV, 5MS9, 5MSZ, 5MTD, 5MUU, 5MV1, 5MX0, 5MY5, 5MY7, 5MZV, 5N0K, 5N40, 5N5Y, 5N6U, 5N6W, 5N8K, 5NB8, 5NDL, 5NFD, 5NHX, 5NII, 5NJG, 5NL7, 5NLT, 5NMU, 5NO7, 5NSC, 5NSJ, 5NTB, 5NV6, 5NX2, 5NX3, 5NXB, 5NZH, 5NZY, 5O2U, 5O46, 5O57, 5O66, 5O6A, 5OAR, 5OBT, 5OC2, 5OCK, 5OMT, 5OOL, 5OP0, 5OQZ, 5OR7, 5OUO, 5SYD, 5T0M, 5T1K, 5T1L, 5T1M, 5T2Q, 5T3V, 5T4M, 5T6T, 5T6V, 5T90, 5T96, 5T9T, 5TA0, 5TGI, 5TH2, 5TS4, 5TS9, 5TTD, 5TV5, 5TVR, 5TWV, 5TX8, 5TXC, 5TXE, 5TYI, 5TZN, 5U06, 5U1Q, 5U25, 5U5F, 5U5M, 5U6A, 5U81, 5U87, 5U9M, 5UBJ, 5UBL, 5UBP, 5UD7, 5UDB, 5UDG, 5UG3, 5UG5, 5UHX, 5UIW, 5UJD, 5UJG, 5UK5, 5UQY, 5USI, 5UUS, 5UV8, 5UYT, 5UZ4, 5V0M, 5V13, 5V2O, 5V3M, 5V50, 5V6I, 5V8E, 5V8F, 5VAT, 5VAV, 5VB9, 5VC7, 5VG2, 5VKJ, 5VKV, 5VLH, 5VLI, 5VLL, 5VMR, 5VO0, 5VOB, 5VOD, 5VR1, 5VU2, 5VXZ, 5VZ4, 5W0Y, 5W21, 5W57, 5W78, 5W7A, 5W8Y, 5W9F, 5WA2, 5WB7, 5WB8, 5WCO, 5WCV, 5WDA, 5WGW, 5WHT, 5WHU, 5WLS, 5WM0, 5WN9, 5WNA, 5WRR, 5WSI, 5WSN, 5WTL, 5WUP, 5WUR, 5WXF, 5WXO, 5X0S, 5X0T, 5X1Y, 5X3H, 5X47, 5X4R, 5X4S, 5X55, 5X5S, 5X5V, 5X6S, 5XAN, 5XBP, 5XBU, 5XCC, 5XCO, 5XF0, 5XGL, 5XIV, 5XJX, 5XKX, 5XLL, 5XM3, 5XM4, 5XMG, 5XMZ, 5XN3, 5XNU, 5XO3, 5XOM, 5XOZ, 5XPB, 5XPV, 5XPW, 5XQ3, 5XS1, 5XSY, 5XTA, 5XWQ, 5XYM, 5Y0R, 5Y0W, 5Y10, 5Y32, 5Y7L, 5Y9W, 5YAG, 5YDJ, 5YGG, 5YJG, 5YO3, 5YQ0, 5YQ5, 5YSC, 5YU5, 5YXW, 5Z11, 5Z1Y, 5Z3G, 5Z5K, 5Z5R, 5ZBI, 5ZCN, 5ZE3, 5ZET, 5ZFV, 5ZGB, 5ZJI, 5ZJL, 5ZNG, 5ZO1, 5ZU5, 5ZU6, 5ZX8, 6A2J, 6A56, 6A69, 6ADQ, 6AGF, 6ANY, 6APL, 6AR7, 6ATK, 6ATS, 6AV8, 6AVE, 6AX2, 6AZ1, 6B5W, 6B7N, 6BA3, 6BAQ, 6BBK, 6BBP, 6BDJ, 6BE7, 6BE9, 6BEN, 6BEO, 6BER, 6BES, 6BET, 6BEU, 6BEW, 6BL9, 6BLH, 6BMM, 6BRB, 6BSU, 6BTM, 6BVH, 6BVU, 6BVW, 6BVX, 6BVY, 6BX9, 6BXB, 6BXO, 6C23, 6C24, 6C3R, 6C5V, 6CD2, 6CEG, 6CEI, 6CF8, 6CFB, 6CKU, 6CMO, 6CPD, 6CPL, 6CPS, 6CSY, 6CV7, 6CWS, 6CZG, 6D03, 6D2C, 6D3X, 6D3Y, 6D7A, 6D8C, 6DCQ, 6DDE, 6DDR, 6DHL, 6DKZ, 6DL0, 6DL1, 6DS5, 6DTJ, 6DXX, 6E0D, 6E3Y, 6E62, 6E7K, 6E7R, 6E8N, 6EA5, 6EDQ, 6EFY, 6EGT, 6EHI, 6EHZ, 6EI6, 6EIB, 6EJF, 6EKE, 6EKH, 6ENA, 6EPK, 6EPY, 6ERC, 6ERX, 6EY3, 6F0W, 6F5D, 6F5G, 6F5P, 6F72, 6F74, 6F7E, 6F8L, 6F99, 6F9G, 6FB4, 6FBR, 6FGJ, 6FGM, 6FI2, 6FJ2, 6FKM, 6FM5, 6FON, 6FOT, 6FOU, 6FOV, 6FPF, 6FPG, 6FWN, 6G1B, 6G7O, 6GB1, 6GCS, 6GDG, 6GEJ, 6GFB, 6GH8, 6GIQ, 6GJ1, 6GJE, 6GK3, 6GM2, 6GQ4, 6GRF, 6GRT, 6GT7, 6H2T, 6H41, 6H7D, 6H7K, 6HBA, 6HDS, 6HKC, 6HMA, 6HN9, 6HVB, 6HVC, 6I0Y, 6IJE, 6ISA, 6IU9, 6IUJ, 6IUT, 6IWQ, 6IWR, 6MAG, 6MEI, 6MLU, 6MS4, 6MUI, 6MUS, 6MYG, 6N25, 6N29, 6N7Q, 6N9T, 6NBN, 6NLP

References

[1] Cantarella, J.; Duplantier, B.; Shonkwiler, C.; Uehara, E. A fast direct sampling algorithm for equilateral closed polygons. *Journal of Physics A: Mathematical and Theoretical* **2016**, *49*, 275202.

[2] Dabrowski-Tumanski, P.; Niemyska, W.; Pasznik, P.; Sulkowska, J.I. LassoProt: server to analyze biopolymers with lassos. *Nucleic acids research* **2016**, *44*, W383–W389. 10.1093/nar/gkw308.
